# A mechanistic account of visual discomfort

**DOI:** 10.3389/fnins.2023.1200661

**Published:** 2023-07-20

**Authors:** Olivier Penacchio, Xavier Otazu, Arnold J. Wilkins, Sarah M. Haigh

**Affiliations:** ^1^Department of Computer Science, Universitat Autònoma de Barcelona, Bellaterra, Spain; ^2^Computer Vision Center, Universitat Autònoma de Barcelona, Bellaterra, Spain; ^3^School of Psychology and Neuroscience, University of St Andrews, St Andrews, United Kingdom; ^4^Department of Psychology, University of Essex, Colchester, United Kingdom; ^5^Department of Psychology, University of Nevada Reno, Reno, NV, United States; ^6^Institute for Neuroscience, University of Nevada Reno, Reno, NV, United States

**Keywords:** visual discomfort, visual stress, interindividual differences, natural scenes, efficient coding, hypermetabolism, urban scenes, computational modelling

## Abstract

Much of the neural machinery of the early visual cortex, from the extraction of local orientations to contextual modulations through lateral interactions, is thought to have developed to provide a sparse encoding of contour in natural scenes, allowing the brain to process efficiently most of the visual scenes we are exposed to. Certain visual stimuli, however, cause visual stress, a set of adverse effects ranging from simple discomfort to migraine attacks, and epileptic seizures in the extreme, all phenomena linked with an excessive metabolic demand. The theory of efficient coding suggests a link between excessive metabolic demand and images that deviate from natural statistics. Yet, the mechanisms linking energy demand and image spatial content in discomfort remain elusive. Here, we used theories of visual coding that link image spatial structure and brain activation to characterize the response to images observers reported as uncomfortable in a biologically based neurodynamic model of the early visual cortex that included excitatory and inhibitory layers to implement contextual influences. We found three clear markers of aversive images: a larger overall activation in the model, a less sparse response, and a more unbalanced distribution of activity across spatial orientations. When the ratio of excitation over inhibition was increased in the model, a phenomenon hypothesised to underlie interindividual differences in susceptibility to visual discomfort, the three markers of discomfort progressively shifted toward values typical of the response to uncomfortable stimuli. Overall, these findings propose a unifying mechanistic explanation for why there are differences between images and between observers, suggesting how visual input and idiosyncratic hyperexcitability give rise to abnormal brain responses that result in visual stress.

## Introduction

Some static images, particularly stripes, are consistently reported as aversive, causing headaches, eyestrain, perceptual distortions, hallucinatory colours or shapes, a series of bodily symptoms regrouped under the term visual stress (Wilkins, 1995). Stripes can also trigger seizures in individuals with photosensitive epilepsy (Wilkins et al., 1984; Radhakrishnan et al., 2005; Hermes et al., 2017). The current literature considers visual stress as resulting from unusually strong neural activity in the visual cortex (Haigh et al., 2013). Visual stress occurs in a range of neurological conditions that are co-morbid with epilepsy (Wilkins et al., 2022), consistent with the theory that cortical hyperexcitability underlies visual stress. In migraine, hyperexcitability is also suggested by a large haemodynamic response to unpleasant sensory stimuli, a low phosphene threshold in response to transcranial magnetic stimulation, and the use of antiepileptics in migraine prophylaxis (Porciatti et al., 2000; Huang et al., 2003; Ferrari et al., 2015; Mulleners et al., 2015). In the non-clinical population, images that trigger visual stress are generally associated with a large cortical haemodynamic response, as measured using functional magnetic resonance imaging (Huang et al., 2003, 2011) and near infra-red spectroscopy (Haigh et al., 2013, 2015; Le et al., 2017), or a large electrophysiological response, for example, steady-state evoked potentials (O'Hare, 2016; Haigh et al., 2019; Gentile and Aguirre, 2020; Lindquist et al., 2021) and event-related potentials (Haigh et al., 2019).

To date, no general principle has explained the link between the spatial structure of an image and the visual stress associated with it, but the most satisfying account for the relationship between visual stress and enhanced cortical activity comes from a prevalent theory in sensory neuroscience, that of efficient coding (Attneave, 1954; Barlow, 1961). The theory of efficient coding states that sensory systems have evolved under the selective pressure to provide an efficient representation of the stimuli relevant for survival in an organism’s environment. The efficiency is expressed both in terms of the information transmitted and the metabolic cost of the neural processes involved. The theory has received strong empirical and theoretical support for different sensory modalities (Olshausen and Field, 1996; Lewicki, 2002; Machens et al., 2005). A large body of theoretical and empirical evidence has demonstrated an accord between neural encoding and regularities in the relevant natural environment (e.g., Olshausen and Field, 1996; Lewicki, 2002; Machens et al., 2005). An important regularity concerns how the correlation between the luminance of two nearby locations in a natural scene decreases with distance. It is encapsulated in the Fourier power spectrum of natural scenes, which decreases with spatial frequency f as $1/f^{\alpha }$, with an exponent α consistently found between 1.6 and 2.4 (Field, 1987; Tolhurst et al., 1992; Geisler, 2008). In agreement with the theory of efficient coding, discrimination performance is optimal for stimuli with such statistics (Knill et al., 1990; Parraga et al., 2000; Geisler et al., 2001). Similarly, measuring the deviation from $1/f^{\alpha }$ provides a reliable predictor of the visual discomfort a visual stimulus provokes (Fernandez and Wilkins, 2008; Juricevic et al., 2010; O'Hare and Hibbard, 2011; Penacchio and Wilkins, 2015; Ogawa and Motoyoshi, 2020), and the way in which the chromaticity differences in a stimulus depart from those in nature predicts visual discomfort (Penacchio et al., 2021).

Whilst the theory of efficient coding provides a theoretical foundation for the understanding of visual stress, measuring deviations with respect to the statistical regularities of natural scenes can only provide *functional* models (Carandini and Heeger, 2012; Kay, 2018) of visual stress, namely models that describe a functional relationship between input, here static images, and output, here observers’ self-reported discomfort when viewing the images. Understanding *mechanistically* how the spatial structures of static images cause discomfort therefore remains a major challenge. In a first computational attempt to characterize the neural correlate of visual discomfort using a mechanistic model (Hibbard and O'Hare, 2015), computed the activity of a bank of simple cells in response to natural images and to sine gratings, a class of images associated with visual discomfort. They found that the response of the model to natural images was sparser than that to gratings, involving fewer “neurons” and therefore consistent with the excessive metabolism associated with uncomfortable images. The approach, however, had three limitations. (1) The study considered only two extreme classes of stimuli, natural images, and gratings, and not more complex real-life scenes. (2) The model did not include contextual influences, i.e., the way the activity of a neuron is modulated by the activity of neighbouring neurons (Fitzpatrick, 2000; Carandini et al., 2005). Contextual modulations in the early visual system are fundamental to understand how the strength of activity of a neuron can be deeply modified depending on the spatial structure of the input outside its classical receptive field (Zipser et al., 1996; Vinje and Gallant, 2000). (3) The model proposed in (Hibbard and O'Hare, 2015) could not account for differences between individuals, for example by considering individual differences in the balance between cortical excitation and inhibition, a possible reason for increased susceptibility to discomfort in some individuals (Adjamian et al., 2004).

In this study, we systematically analyzed the activity of a biologically based neurodynamic model of the early visual cortex that implements contextual influences in response to images from urban landscapes and abstract art. We considered theories of visual coding that link image spatial structure and brain activation in order to investigate how different metrics of the model activity predicted observers’ self-reported visual discomfort. Our aim was twofold: (1) To gain a mechanistic understanding of the differences in induced discomfort between images, and (2) to investigate possible mechanistic explanations for individual differences in susceptibility to discomfort. We first identified possible neural markers of discomfort. We next analyzed how these markers changed when the ratio of excitation over inhibition in the network was modified. This allowed us to test the putative role of disparities in balance between cortical excitation and inhibition in the mechanisms underlying inter-individual differences in susceptibility to visual discomfort.

## Materials and methods

### Stimuli, participants, and procedure

We considered four sets of stimuli previously used in the literature, two sets of photographs of urban architecture (building frontages) and two sets of images of abstract art.

#### Architecture stimuli

We used two sets, hereafter, Architecture 1 and 2, *N* = 74 images in each, previously considered in a study on the contribution of spatial frequencies to visual discomfort for which the experiments were held in person (Penacchio and Wilkins, 2015). Participants [*N* = 10 in each set, see (Penacchio and Wilkins, 2015)] viewed each colour 720 × 960-pixel images for an unlimited time on a calibrated LCD display with a size of 20 cm x 27 cm (width x height) at 80 cm, representing a visual angle of 15°. They were instructed to rate the images for discomfort on a Likert scale, from 1 for ‘not uncomfortable at all’ to 7 for ‘very uncomfortable’. We explicitly stated that we were interested in discomfort, which we assumed to be distinct from unpleasantness, ugliness, or un-preference.

#### Art stimuli

We considered two sets of images of abstract art from a study on the contribution of variations in chromaticity to visual discomfort (Penacchio et al., 2021), Art 1 and 2, *N* = 50 images in each. For these sets, the responses were collected online using Qualtrics, in agreement with COVID-19 protocols. Participants (Art 1, *N* = 53 after 5 were discarded because they showed no variability in their responses, 37 female, 2 non-binary, 14 male, mean age 20.0 years, SD age 2.0; Art 2, *N* = 79 after 1 was discarded on the same grounds as for Art 1, 59 female, 6 non-binary, 14 male, mean age 20 years, SD age 2.5) viewed each grey-level 512×512 pixel image for an unlimited duration and had to report their level of discomfort on a Likert scale (1, ‘no discomfort’, 2, ‘some discomfort’, 3, ‘moderate discomfort’, 4, ‘uncomfortable, and 5, ‘very uncomfortable’). As these two sets were rated online, we had no control on the viewing conditions, and therefore no knowledge of the visual angle represented by the stimuli for each participant. All participants were recruited from the University of Nevada, Reno, and consented to the study electronically. They obtained a course credit for their time. All verified that they had normal or corrected to normal vision and none reported a diagnosed psychiatric or neurological condition. The protocol was approved by the Institutional Review Board at the University of Nevada, Reno (333057), and was conducted in accordance with the Declaration of Helsinki.

Supplementary File “Raw data” (Penacchio et al., 2023) provides a visualisation of all the ratings of all observers for the four sets of stimuli. While the sets of urban scenes rated for discomfort consisted of colour images, these images were modestly coloured and colour differences did not explain variance in discomfort (Penacchio et al., 2021; see Electronic Supplementary Material).

### Experiments

**Figure 1:**
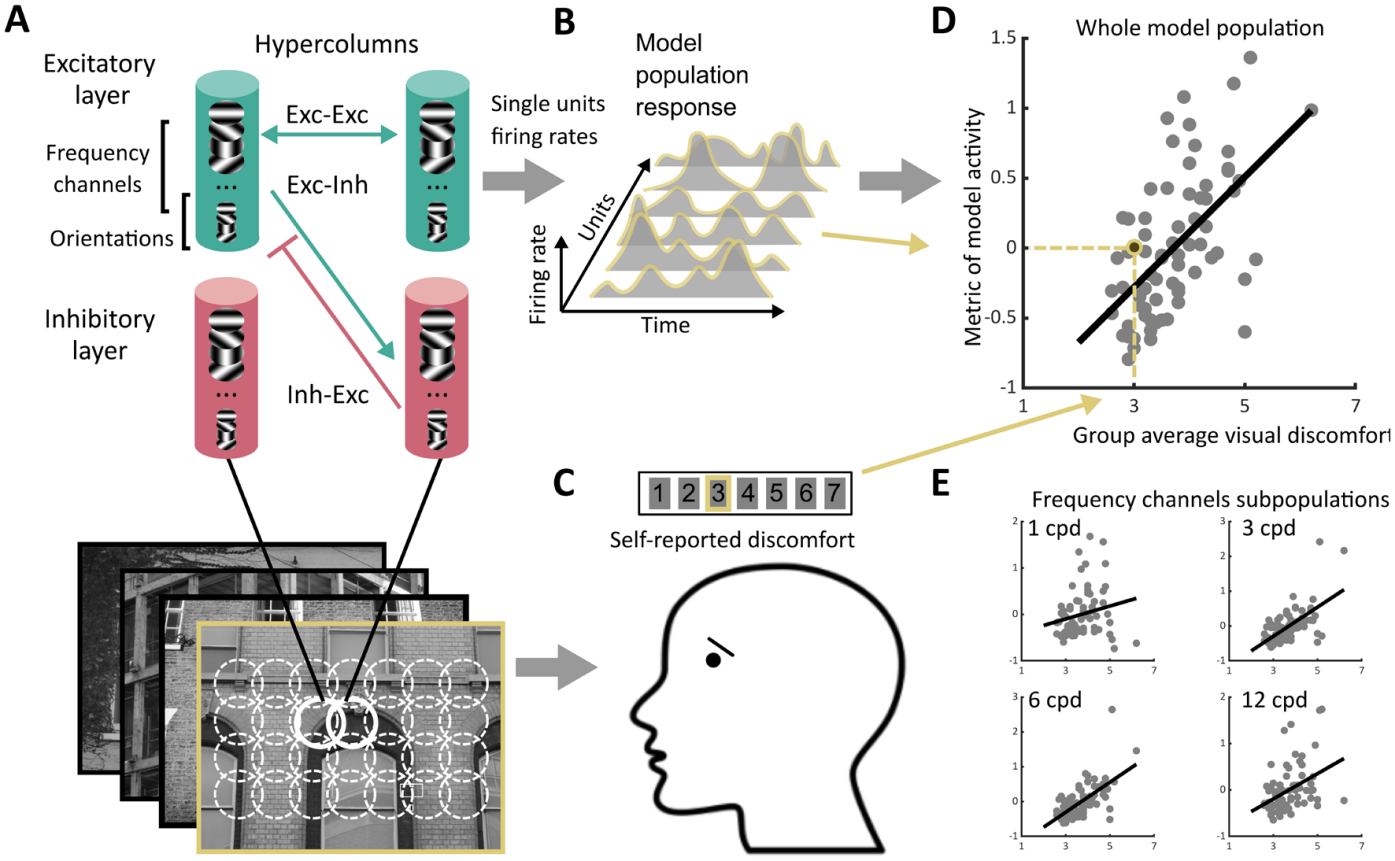
Schematics of the experiment. **(A)** We processed images from four sets of stimuli (Architecture 1 and Architecture 2, *N* = 74 each, Art 1 and Art 2, *N* = 50 each) using a neurodynamic model of the early visual cortex. This model consisted of a layer of excitatory units and a layer of inhibitory units scattered in a grid of hypercolumns organised retinotopically, each including units sensitive to luminance edges with different spatial orientations and spatial frequencies. The hypercolumns were interconnected through excitatory-excitatory, excitatory-inhibitory, and inhibitory-inhibitory connections following a biologically plausible pattern of lateral connections. For each image, we recorded the firing rates of all the excitatory units in the model over several temporal iterations of the model, leading to **(B)** the model population response to the image, i.e., vectors of non-negative numbers. **(C)** Observers reported perceived discomfort when viewing each image in one of the four sets of stimuli by rating each stimulus on a Likert scale in which the lowest value meant ‘not uncomfortable at all’ and the highest ‘very uncomfortable to look at’. **(D)** We then regressed (here, for illustration, group average of) reported visual discomfort against different metrics of the model population response. The metrics were chosen to reflect three main hypotheses on the neural correlate of visual discomfort (see section Metrics of model activity, Rationale for types of metrics considered). **(E)** To analyse the contribution to visual discomfort of different spatial frequencies, we also regressed reported visual discomfort against the same metrics applied to the subset of units in the model sensitive to a given spatial frequency (‘frequency channels’, see Methods). The subpanels only show four of the twelve channels.

Our study consisted of two experiments. In Experiment 1, we looked for possible markers of visual discomfort in the activity of a dynamic model of the visual system. Therefore, we contrasted participants’ self-reported discomfort when viewing an image to different metrics of the activity of a model of the early visual system in response to this image (see Figure 1). We found such markers, and so in Experiment 2 we explored whether and how they would change when the ratio of excitation over inhibition was altered in the model. Since we could not measure intra-cortical inhibition, Experiment 2 was entirely computational.

### Model of the early visual cortex

#### Model architecture

The model was made of two components. Component 1 consisted of units whose receptive fields (RFs) mimic those of simple cells in the primary visual cortex (V1) and have been shown to fit well with physiological data (Serre and Riesenhuber, 2004; Serre et al., 2007). Component 2 was a network that takes the output of Component 1 and implements contextual influences. It consisted of a firing-rate neurodynamic network made of units that emulate (‘glutamatergic’) excitatory and (‘GABAergic’) inhibitory neurons connected as a recurrent network that emulates the connectivity of the visual cortex underpinning contextual influences (Wilson and Cowan, 1972; Li, 1999, 2002; Dayan and Abbott, 2001; Figure 1A).

The units in Component 1 were modelled using Gabor filters sensitive to four regularly spaced orientations (0°, 45°, 90° and 135°) and 12 spatial frequencies, corresponding to 12 regularly spaced wavelengths, ranging from 1 cycle per degree (cpd) to 10.8 cpd for the experimental conditions of Architecture 1 and 2. The output of the convolution of these filters with the images was the input to the network (Component 2).

The cortical columns (hypercolumns) in Component 2 densely sampled the input image (one hypercolumn per pixel, resulting in a total of 256^2^ hypercolumns) (Figure 1A). The population of excitatory units within each hypercolumn included all 48 types of simple cells (4 orientations x 12 spatial scales). The hypercolumns also included a ‘mirror’ population of inhibitory units. Excitatory cells were connected to each other through monosynaptic excitatory connections, and disynaptically via inhibitory interneurons [conforming to Dale’s law stating that neurons in the cortex are either excitatory or inhibitory (Eccles, 1976)]. The architecture and connectivity of the network has been presented in previous work (Li, 1999, 2002; Penacchio et al., 2013) and mimics the biological architecture responsible for contextual modulations of the activity of a neuron by stimulation of its non-classical receptive field (nCRF). It is based on lateral connections that simulate the connectivity in mammal and primate early visual cortex (Knierim and Vanessen, 1992; Kapadia et al., 1995; Weliky et al., 1995). In particular, the connectivity is set up such that: (i) mutual monosynaptic excitation is strong between neighbouring units sensitive to similar spatial frequencies and to orientations similar to the direction formed by these units [a cortical feature at the basis of contour enhancement (Li, 1999)], and, (ii) inhibition is strong between neighbouring units sensitive to orientations perpendicular to the orientation these units form [a property thought to be at the basis of iso-orientation suppression (Li, 1999)]. Overall, the activity of a unit is driven by the input within its CRF and modulated by contextual influence in its nCRF. Modulation can be suppressive or facilitatory, depending of the strength of horizontal recruitment, but tends to be more suppressive for higher input levels (see Li, 1999) in agreement with empirical observations (Hirsch and Gilbert, 1991; Weliky et al., 1995; Cavanaugh et al., 2002).

The architecture of Component 2 has been previously shown to be able to account for figure-ground segmentation, bottom-up saliency, contour grouping, and brightness induction (Zhaoping and May, 2007; Zhang et al., 2012; Penacchio et al., 2013; Zhaoping and Zhe, 2015; Berga and Otazu, 2020, 2022). We also verified that simulating the nCRF of units with naturalistic stimuli increased the sparseness of their response (Vinje and Gallant, 2000; Haider et al., 2010; Supplementary Material S2; Penacchio et al., 2023).

Importantly, as our main purpose in this paper was to understand whether a biologically realistic model of the early visual cortex processes uncomfortable images differently from other images, we did not fit any parameters in any of the two components of the model but used the previously published parameters [Component 1, (Serre and Riesenhuber, 2004, Serre et al., 2007); Component 2 (Li, 1999; Penacchio et al., 2013)]. Supplementary Material S1 gives a full description of the two components of the model including the equations that govern the dynamic of the network. Note that our choice for the size of the input images (256 × 256) imposed an upper limit to the spatial frequencies handled by the model (the highest frequency was 10.8 cpd). This choice reflected both a constraint on the computational load for processing all the images with all the possible values of gain (see below) and robust evidence from the literature that the frequencies that contribute most to discomfort are below 10 cpd (Wilkins et al., 1984; O'Hare and Hibbard, 2011).

#### Model dynamic and output

The dynamic of the model was simulated using a discrete time implementation with an Euler integration scheme of the first order (Strogatz, 2015). The model assumes a periodic activity (‘steady state’) after 3 to 4 membrane time constants after stimulus onset (Li, 1999; Penacchio et al., 2013). For each image, the output of the model, hereafter, *model population response*, was the vector $\left(x_{is\theta }\left(t\right)\right)$ formed by the firing rates of all the excitatory cells between the 4th and 20th membrane time constants, where $i\in {\left[1,256\right]}^{2}$ is the hypercolumn position, s∈[1,12] is the frequency channel, θ∈{0°,45°,90°,135°} is the orientation, and t∈{4,5,…,20} is the membrane time constant, giving a vector of length $\approx 5.35\times 10^{7}$ for each image (Figure 1B). All metrics to analyse the activity of the model were based on this vector.

#### Input pre-processing

Before being processed by the model, the images were cropped to centred squares of maximal size, then converted to luminance using the measurement of the display’s R, G and B channels [Architecture 1 and 2, see Penacchio and Wilkins (2015)], or Matlab’s (MATLAB, 2019) *rgb2gray* function (Art 1 and 2), and finally down-sampled to 256 × 256 pixel images using Matlab’s function ‘*imresize*’ with a nearest neighbour algorithm. To avoid edge effects, the images were padded using a standard mirroring process of width 28 pixels, resulting in 312 × 312-pixel images input to the model. Only the 256 × 256 central positions were considered for the model population response$\;\left(x_{is\theta }\left(t\right)\right)$.

#### Excitation/inhibition balance

We manipulated the ratio of excitation over inhibition (E/I) in the model (e.g., modelling a lack of Gamma-aminobutyric acid (GABA) neurotransmitter) by decreasing the strength of activation of the inhibitory layer of the starting model (hereafter, *reference model*) using a multiplicative factor called gain. The gain varied between 1 (no modification, reference model) to 0 (no inhibition at all) by steps of −0.125. See Supplementary Material S1 for details.

### Rationale for using a neurodynamic model

We believe that many static models of early spatial vision that include units sensitive to different spatial frequencies and orientations, non-linearity and divisive normalization to implement contrast gain-control (Heeger, 1992; Foley, 1994; Cavanaugh et al., 2002; Kay et al., 2013; Schutt and Wichmann, 2017) would also be good model candidate to predict visual discomfort. We chose to use instead an excitatory-inhibitory neurodynamic model based on Zhaoping Li’s seminal model (Li, 1999, 2002) for several reasons. First, this allowed us to have separate excitatory and inhibitory populations, and therefore to test putative hypotheses on the role played by the relative strength of excitation and inhibition in visual discomfort, thought to play a central role in individual differences in susceptibility to visual discomfort. Second, this allowed us to inherit from (Li, 1999, 2002) a realistic implementation of mutual influences between neighbouring units tuned to different spatial orientations. Third, including timing goes a step further toward more complex models that in the future will allow a careful estimation of the amount of activity in the gamma band, which are crucial to understand photosensitive epilepsy (e.g., Hermes et al., 2017 and see Discussion).

### Metrics of model activity

#### Rationale for types of metrics considered

Two main theories of visual coding link brain activation and image structure. The contrast energy theory states that activation in the primary cortex depends on local contrast at different spatial frequencies, independently of the spatial arrangement of the luminance edges producing this contrast (Movshon et al., 1978; Albrecht and Hamilton, 1982; Rieger et al., 2013). The theory of sparse coding, by contrast, supposes that the early visual system has adapted to process the specific arrangements and redundancy of local contrast features found in natural scenes with a reduced number of neurons strongly active simultaneously (Atick and Redlich, 1992; Field, 1994; Olshausen and Field, 1996, 2004; Vinje and Gallant, 2002; Hyvarinen et al., 2009), saving metabolic energy. We therefore considered metrics of the model population activity in response to each image $\left(x_{is\theta }\left(t\right)\right)$ related to (1) the *model population activation level* (‘total amount of firing of all units when processing the image’) and (2) the *sparseness of the model response* (‘to what extent the encoding of the image was carried out by a small number of active units across retinotopic space and time’). We also considered a metric based on (3) the *isotropy of the model response* (‘how much activity was evenly distributed across orientations in the model hypercolumns’). Our rationale was that periodic patterns such as stripes, causing a concentration of neural activity in a subpopulation of cortical cells sensitive to the same orientation, are strong inducers of pattern-sensitive epilepsy and visual discomfort (Wilkins et al., 1979, 1984; Meldrum and Wilkins, 1984; O'Hare and Hibbard, 2011; Hermes et al., 2017).

#### Metrics implementation

**Figure 2:**
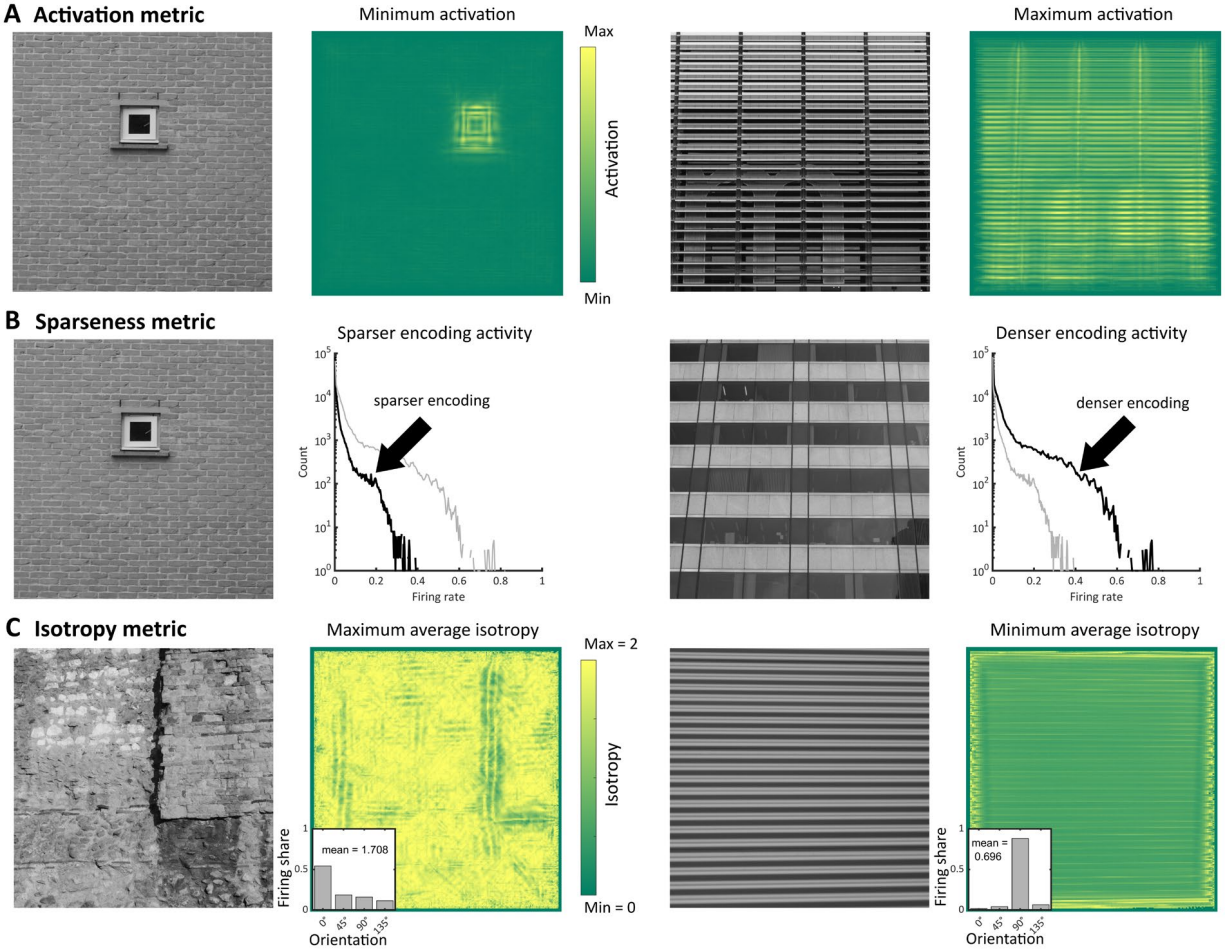
Illustration of the metrics used as markers of visual discomfort. **(A)** Activation level of model population response: (from left to right, first panel) Image in the set Architecture 1 with the lowest average activation level, (second) corresponding heatmap of activation summed over all membrane times, frequency channels and orientations, (third) image with the highest average activation level, and (fourth) corresponding heatmap of activation summed as in the second panel; in the heatmaps, the yellower the colour the higher activation. **(B)** Sparseness of model population response: (first panel) Image with the highest value for the sparseness metric in the same set as in panel **(A)**, (second, black curve) corresponding histogram of firing rates and (grey curve) histogram for the image in the third panel for comparison, (third) image with the lowest value for the sparseness metric, and (fourth, black curve) corresponding histogram of firing rates. **(C)** Isotropy metric: (first panel) Image with the highest level of the metric in the same set, (second) heatmap of isotropy averaged across all membrane time and frequency channels and (inset) example of distribution of responses across orientations with isotropy equal to the average for the whole image (1.708), (third) Image with the lowest level of the metric, and (fourth) heatmap of isotropy averaged as in panel two and (inset) example of distribution with isotropy equal to the average of the whole image (0.696).

(1) The model activation level, similar to a measure of contrast energy, was measured using the $L^{1}$-norm of the model population response, $E\left(\left(x_{is\theta }\left(t\right)\right)\right)=∥\left(x_{is\theta }\left(t\right)\right)∥{}_{1}=\sum_{i,\theta ,s,t}\left|x_{is\theta }\left(t\right)\right|$, where (i,θ,s,t) runs over all the hypercolumns, orientations, frequencies and membrane time constants considered. As the choice of a particular norm over others is arbitrary, we also measured the activation level using alternative metrics (see Supplementary Material S3; Penacchio et al., 2023). (2) The sparseness of the model population response was measured as $S\left(\left(x_{is\theta }\left(t\right)\right)\right)=-\sum_{i,\theta ,s,t}\tanh{\left|x_{is\theta }\left(t\right)\right|}^{2}\;$(Hyvarinen, Hurri et al., 2009). We also computed alternative measures of sparseness (see Supplementary Material S3; Penacchio et al., 2023). (3) Isotropy was computed using the (Shannon) entropy $H_{ist}$ of the distribution of response for each hypercolumn i, spatial frequency s and membrane time t, and taking the grand mean, $H\left(\left(x_{is\theta }\left(t\right)\right)\right)\;$= $\sum_{i,s,t}H_{ist}/N$, where $N=256^{2}\times 12\times 16$ is the number of terms in the sum, and $H_{ist}=-\sum_{\theta =0{}^{\circ},45{}^{\circ},90{}^{\circ},135{}^{\circ}}p_{\theta }\left(i,s,t\right)\log_{2}\left(p_{\theta }\left(i,s,t\right)\right)$ is the entropy of the probability distribution obtained by normalising the vector ($x_{is0{}^{\circ}}\left(t\right),x_{is45{}^{\circ}}\left(t\right),x_{is90{}^{\circ}}\left(t\right),x_{is135{}^{\circ}}\left(t\right)$) so that it sums to one. Entropy measures isotropy in the sense that if all the orientations are equally represented at (i,s,t), (i.e., $x_{is0{}^{\circ}}\left(t\right)=x_{is45{}^{\circ}}\left(t\right)=x_{is90{}^{\circ}}\left(t\right)=x_{is135{}^{\circ}}\left(t\right)$), $H_{ist}$ assumes its maximal value, $H_{ist}=\log_{2}\left(4\right)=2$. When the response to one orientation is greater than that for the others we have $H_{ist}$ < 2, and $H_{ist}=0$ if only a single orientation is represented (as for ‘stripes’). For analysing the frequency channels separately, we applied the same metrics to the subpopulation of units corresponding to each channel, i.e., $\left(x_{is_{0}\theta }\left(t\right)\right)$ with $s_{0}$ being one of the 12 spatial frequencies (Figure 1E). Figure 2 illustrates the computations of the metrics and shows the stimulus with the minimum and maximum value of each metric in set Architecture 1 as well as the relevant aspect of the network activity [see Supplementary File “all markers’ values” (Penacchio et al., 2023) for an illustration of the markers values for all the stimuli in Architecture 1].

### Natural images

To estimate the distribution of isotropy of the model response for natural images we randomly selected 100 images of the van Hateren’s database of calibrated natural images (van Hateren and van der Schaaf, 1998), processed them as done with the stimuli of the experiments and extracted this metric for each input image.

### Statistical analysis

We used linear mixed effects (multilevel) models to analyse possible correlations between metrics and participants’ self-reported discomfort in Experiment 1. In all models, the metrics were considered as fixed effects and participant identity was considered as a random effect (i.e., with random intercept, and, when possible, random slope). This allowed us to estimate the effect of possible markers of discomfort in the general population while ignoring the specificities of each participant, including specific viewing conditions and apparatus for the sets rated online, Art 1 and 2. The models were fitted in R (R Development Core Team, 2020) using the function *lmer* from the package lme4 (Bates et al., 2014). All the metrics were normalised to a mean of 0 and a SD of 0.5 (e.g., Gelman and Hill, 2006). We selected the models using log likelihood, AIC information criterion, and likelihood ratio (BIC is reported for information). For hypothesis testing we used χ^2^-distributions whose degree of freedom were the differences in degrees of freedom between the models to be compared. Following recommended practice (Meteyard and Davies, 2020), all the details about the models tested and adopted are provided in Supplementary Material, section Statistical Inference.

## Results

### Experiment 1: markers of visual discomfort

**Figure 3:**
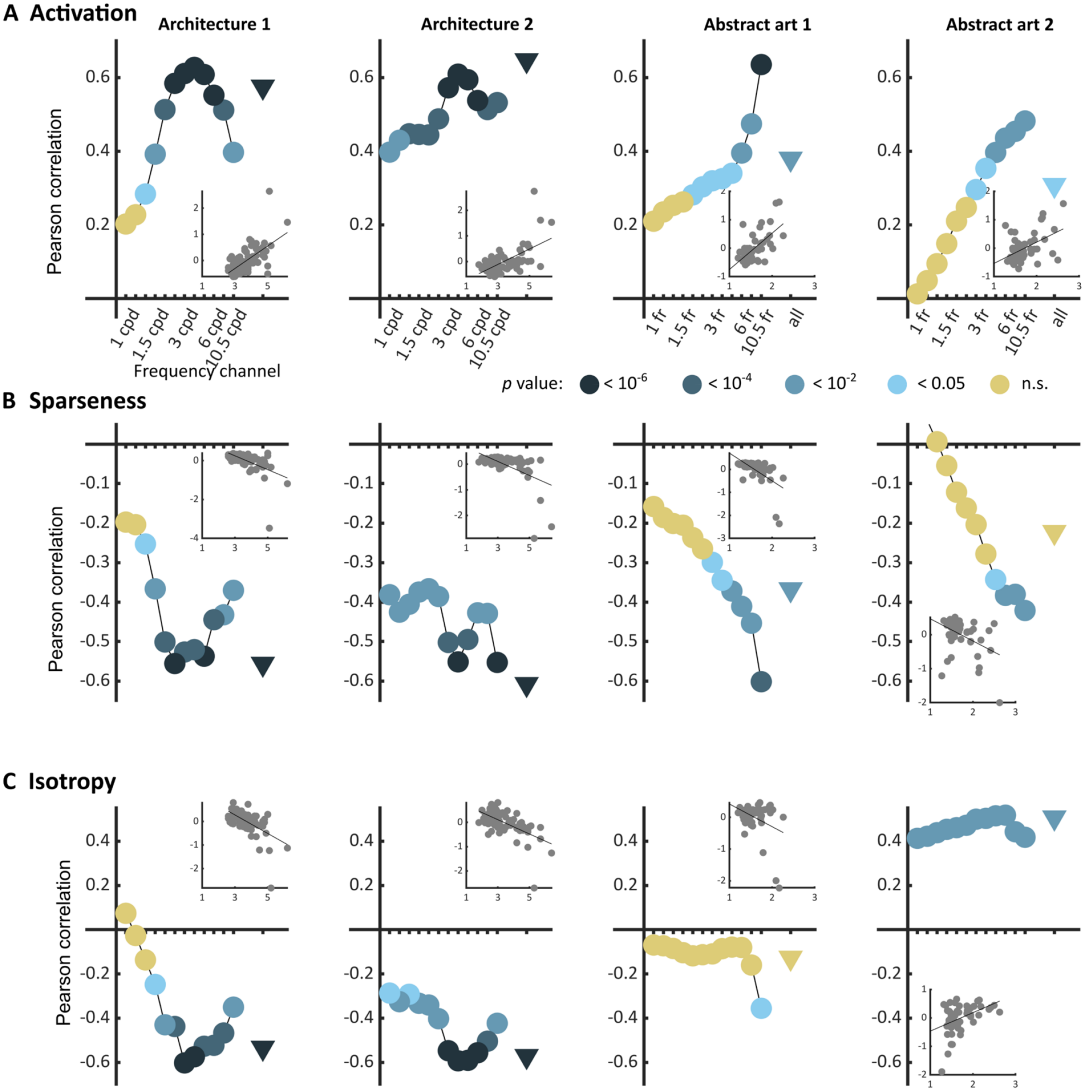
Correlations between average reported visual discomfort against the three main metrics of model population activity, namely **(A)** model population response *activation* level, **(B)** *sparseness* of the model response, and **(C)** *isotropy* in the model response for the four sets of stimuli (from left to right column, Architecture 1, *N* = 74, Architecture 2, *N* = 74, Art 1, *N* = 50, and Art 2, *N* = 50). Each point represents the Pearson’s correlation coefficient for one frequency channel of the model (dots, for the 12 frequency channels of the model) or for the whole model (triangle). The value of *p* of each regression is colour-coded with a level of blue (the darker, the lower the value of *p*), or with yellow for *p*-values above the reference threshold 0.05. Each inset shows the raw data for the regression in the case of the whole population (triangle). All metrics were normalised to a mean value of 0 and standard deviation of 0.5 (see Methods).

**Figure 4:**
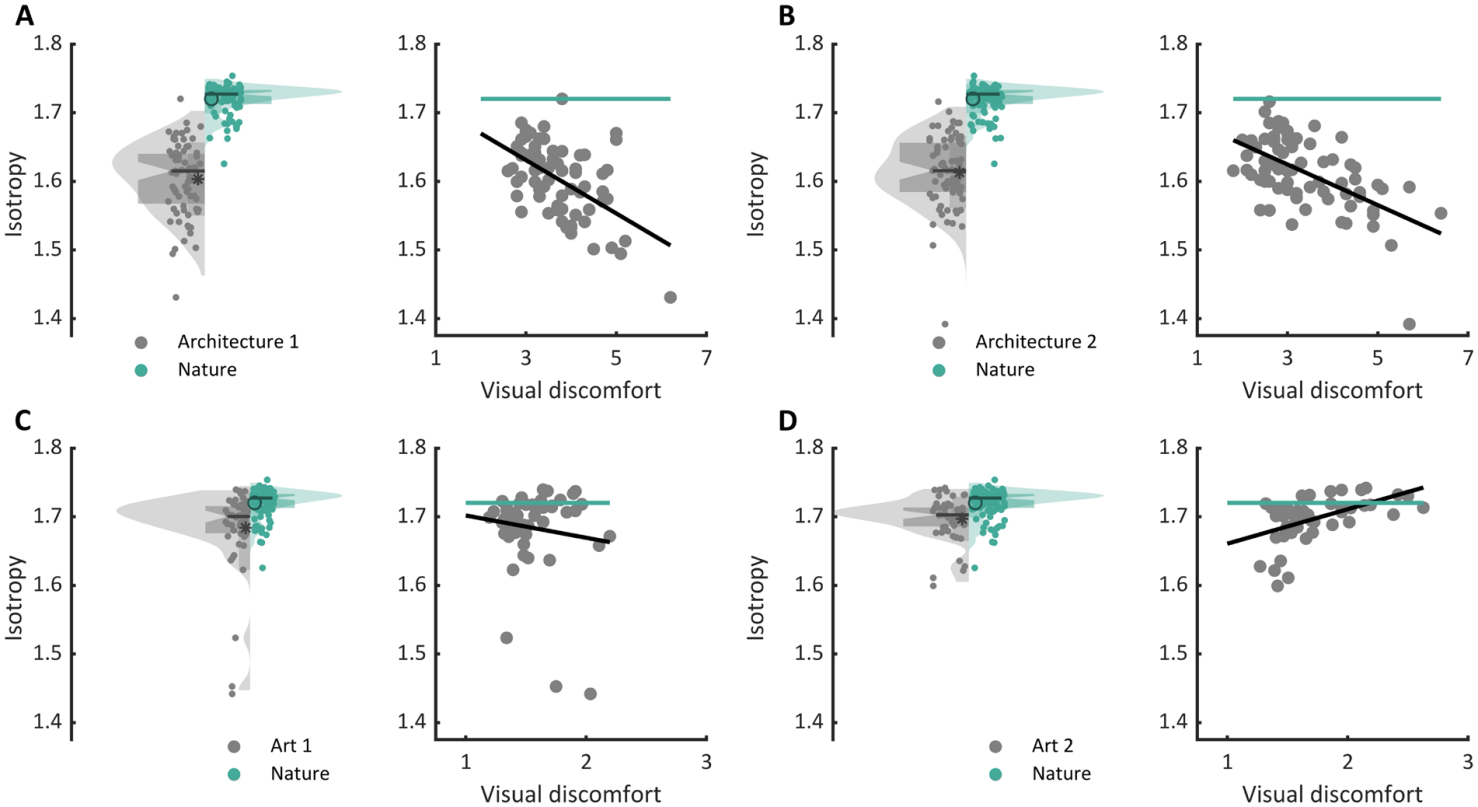
Comparison of the distribution of isotropy in each set with that in nature and regression of isotropy against average reported discomfort for **(A)** the set Architecture 1, **(B)** Architecture 2, **(C)** Art 1, and **(D)** Art 2. In each panel, the left plot shows the distribution of isotropy in the model for all stimuli in the set (grey, left of the central axis) and distribution for a set of natural images (green, right of central axis). The dots show the raw values of the metric for each stimulus (Architecture 1, *N* = 74, Architecture 2, *N* = 74, Art 1, *N* = 50, Art 2, *N* = 50, and natural images, *N* = 100), the box plot show the first (bottom) and third (top) quartile, the notches show the 95% confidence interval for the median, and the star and circle show the mean of the distributions. The right plots in each panel show the regression of isotropy against average reported discomfort (grey) as well as the mean value of the metric for the set of natural images.

The three types of metrics envisioned, activation level, sparseness, and isotropy, were markers of visual discomfort. We found an effect of population activation level on observers’ judgements for the four sets of images. Images that elicited a greater activation in the model were associated with more discomfort (Figure 3A, full model, Architecture 1: *χ*^2^ = 107, df = 3, *p* < 10^−15^; Architecture 2: *χ*^2^ = 157, df = 3, p < 10^−15^; Art 1: χ^2^ = 99, df = 3, *p* < 10^−15^; Art 2: χ^2^ = 78, df = 3, p < 10^−15^). We also found a relationship between the sparseness of encoding of an image and its associated discomfort. Images inducing a less sparse (‘denser’) encoding caused more discomfort (Figure 3B, Architecture 1: χ^2^ = 72, df = 3, *p* < 10^−14^; Architecture 2: χ^2^ = 134, df = 3, p < 10^−15^; Art 1: χ^2^ = 77, df = 3, p < 10^−15^; Art 2: χ^2^ = 43, df = 3, p < 10^−8^). Anisotropy of the model response was clearly associated with discomfort for the two sets showing urban landscapes (Figure 3C, Architecture 1: χ^2^ = 72, df = 3, p < 10^−14^; Architecture 2: χ^2^ = 122, df = 3, *p* < 10^−15^). However, that was not the case for the first set of abstract art (Figure 2C, Art 1: *p* = 0.35), and the relationship was inverted for the second set of abstract art, in which images encoded with a more isotropic activity were associated with more discomfort (Figure 3C, Art 2: χ^2^ = 169, df = 3, p < 10^−15^). To investigate the low predictive power of isotropy for the two sets of art, we compared the distributions of this metric for Art 1 and 2 to that found in natural images. We found that these distributions greatly overlapped those for natural images, as illustrated in Figures 4C,D, left panels (Jensen-Shannon divergence with natural images, 0.265 for Art 1, 0.233 for Art 2), in contrast with the wider distribution and bigger separation with respect to natural scenes in the case or the architectural facades (Figures 4A,B, Jensen-Shannon divergence, 0.855 for Architecture 1, 0.810 for Architecture 2). Accordingly, only a small proportion of the stimuli in Art 1 and 2 had isotropy values that fell outside of the distribution of isotropy found in natural images, values which were associated with discomfort in the sets of urban scenes Architecture 1 and 2.

Table 1 shows the Pearson correlation coefficients of the regressions between metrics and discomfort ratings in Figure 3 (see inset in each panel).

**Table 1:** Pearson correlation coefficients between the three types of metrics and average reported discomfort for the four sets of stimuli.

	Architecture 1	Architecture 2	Art 1	Art2
Activation	**r_E_ = 0.57**, *p* < 10^−7^ ci = [0.40, 0.71]	**r_E_ = 0.65**, *p* < 10^−9^ ci = [0.49, 0.76]	**r_E_ = 0.38**, *p* = 0.0065 ci = [0.11, 0.60]	**r_E_ = 0.31**, *p* = 0.029 ci = [0.03, 0.54]
Sparseness	**r_S_ = − 0.56**, *p* < 10^−6^ ci = [−0.70, −0.38]	**r_S_ = − 0.61**, *p* < 10^−8^ ci = [−0.73, −0.44]	**r_S_ = − 0.37**, *p* = 0.0086 ci = [−0.59, −0.10]	**r_S_ = −0.22**, *ns*, *p* = 0.12; ci = [−0.47, 0.06]
Isotropy	**r_H_ = − 0.53**, *p* < 10^−6^ ci = [−0.68, −0r_E_.35]	**r_H_ = − 0.57**, *p* < 10^−7^ ci = [−0.71, −0.40]	**r_H_ = −0.13**, *ns*, *p* = 0.38 ci = [−0.39, 0.16]	**r_H_ = 0.51**, *p* = 0.00017 ci = [0.27, 0.69]

The values in bold correspond to the correlations that are both significant (under the 0.05 value) and for which the effect’s polarity agrees with the hypothesis of discomfort associated with an overload of activity.

As previous research has shown that the discomfort an image elicits strongly depends on its frequency content (Wilkins et al., 1984; O'Hare and Hibbard, 2011; Hermes et al., 2017), we also considered the frequency channels in the model separately (Figure 3, “frequency channels” in all panels). For the two sets of architecture, we found that visual discomfort was best predicted by the three metrics applied to the channels tuned to spatial frequencies within the range 1.5–6 cpd, as shown by the peaks of correlation around these frequencies in Figures 3A–C for Architecture 1 and 2 (see Supplementary Material S1 for a derivation of the correspondence between frequency channels in the model and visual angle in the experimental conditions for Architecture 1 and 2). These frequencies are those at which human sensitivity is maximal (Campbell and Robson, 1968). By contrast, for the images of art the strength of the correlations between discomfort and the metrics were higher for the channels tuned to the highest frequencies (Figures 3A–C, Art 1 and 2). We believe this difference to reflect the variability in spatial frequencies caused by the uncontrolled nature of the online format in Art 1 and 2.

The three metrics were correlated to each other within each set of stimuli (see Supplementary Figures S2–S5, Supplementary Material S5.1 and Figures S6–S9). However, for the sets of architectural images the best linear combination of the three metrics was a better predictor of visual discomfort than any of the metrics taken separately (Supplementary Material S5), leading to Pearson correlation coefficients of $r_{ESH}$ = 0.63 (*p* < 10^−8^; ci = [0.47, 0.75]) for Architecture 1, $r_{ESH}$ = 0.67 (*p* < 10^−10^; ci = [0.52, 0.78]) for Architecture 2, explaining, respectively, 40 and 45% of the variance in judgements for discomfort. Considering the three metrics together did not provide a better predictor for the sets Art 1 and 2 when the metrics were computed from the full model activity. However, for the frequency channel tuned to the highest spatial frequencies (Figure 3), a linear combination of the metrics predicted better observers’ ratings than any of the metrics in isolation (see Supplementary Material S5.2), with Pearson correlation coefficients of $r_{ESH}$ = 0.39 (*p* = 0.0047; ci = [0.13, 0.61]) for Art 1, $r_{ESH}$ = 0.52 (*p* < 10^−3^; ci = [0.28, 0.70]) for Art 2. Finally, whilst these two metrics are generally correlated, considering activation alongside sparseness, or vice versa, strongly increased model fit for Architecture 1 and Art 2, suggesting a certain degree of independence between them (see Supplementary Material S5.3 and theoretical considerations therein).

### Experiment 2: impact of excitation/inhibition balance on the markers of discomfort

**Figure 5:**
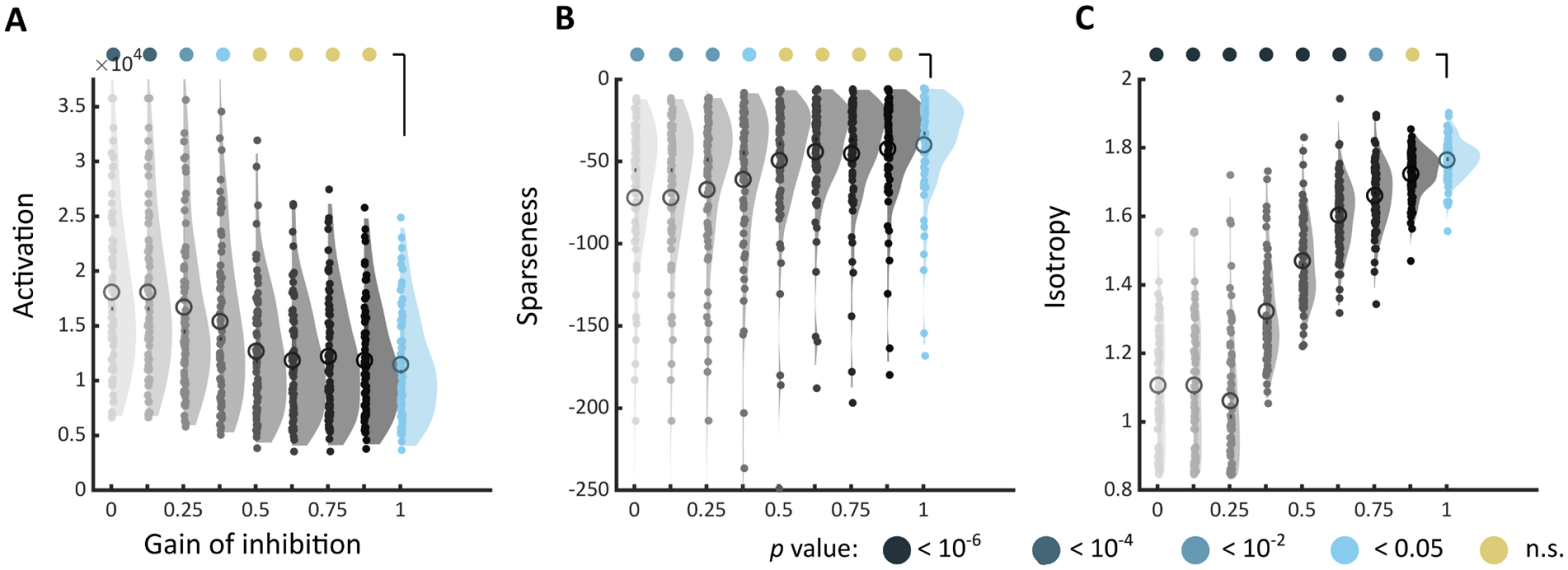
Changes in markers of visual discomfort when the balance of excitation over inhibition is modified. Distributions of **(A)** activation, **(B)** sparseness, and **(C)** isotropy metrics for all the stimuli in Architecture 1 and increasing values of gain for the inhibitory layer. The gain ranged from 0, i.e., no inhibitory activity in the model (top left, light grey distribution), to 1, i.e., reference model (top right, blue distribution), in steps of 0.125. Differences between distributions and the distribution for the reference model were tested using two-sample Kolmogorov–Smirnov tests; p-values are colour coded as in Figure 3. See Supplementary Figures S11–S13 for the equivalent distributions for sets Architecture 2, Art 1 and 2.

When inhibition was decreased in the model, the markers of discomfort identified in Experiment 1 systematically shifted toward values associated with increased discomfort. The shift was toward higher activation level (Architecture 1, used for the sake of illustration, Figure 5A), a less sparse encoding (Figure 5B), and a decreased isotropy in the model response (Figure 5C). These differences with the reference model (gain = 1, top right distribution) reached significance for all metrics (the highest gain leading to a different distribution was gain = 0.375 for the activation level, Kolmogorov–Smirnov test, *D* = 0.297, *p* = 0.0027, and for sparseness, *D* = 0.378, value of *p* <10^−4^, and gain = 0.875 for *D* = 0.351, *p* = 0.00019), showing that the effect of reducing inhibition was stronger for isotropy than for the two other markers. This analysis revealed a similar shift toward values associated with visual discomfort for the three other sets of stimuli, apart from two metrics, activation level and sparseness, for Art 1 (see Supplementary Figures S11–S13).

To evaluate how the changes in the metrics when decreasing inhibition would translate into predicted discomfort, we determined, for each value of gain, the number of images with a metric value above the threshold associated with 15% most discomfort in the original model. To establish this threshold, for each of the three metrics, we used the linear regression of discomfort judgement against the metric using best linear mixed models from Experiment 1 to find the metric value that corresponded to the 15% higher percentile of discomfort ratings. We next computed the number of images beyond this threshold for each value of the gain. We found a systematic increase in the number of images beyond this threshold for decreasing values of gain (see Supplementary Figure S14).

To illustrate how the distribution of firing within hypercolumns changed as the strength of inhibition was decreased, we followed the distribution of the firing activity across orientation at each retinotopic location (averaged over all membrane times and frequency channels) when the gain of inhibition was decreased. For each image, a “winner-takes-all” process took place in the model’s hypercolumns in which all the activity concentrated in a single orientation for low values of inhibition, as if only a single orientation was present in the input stimulus (see Supplementary Figure S15). In other words, as the strength of inhibition was lowered the activity of the model became akin to the activity in response to especially uncomfortable stimuli such as stripes.

## Discussion

In this study, we identified possible markers of visual discomfort in the response of a biologically plausible model of the early visual cortex. Informed by theories in visual neuroscience that link brain activation and image spatial content, and by a growing body of work showing an association between visual discomfort and larger metabolic and electrophysiological response, we derived three types of measures that were candidates for characterising the model’s response to uncomfortable images. We found that the level of activation, the sparseness, and the isotropy of the model population response to images from urban scenes and abstract art were good predictors of the visual discomfort experienced by observers when viewing the images. Our results therefore provide new insights into how overload in the visual system may lead to discomfort by suggesting three possible non-mutually exclusive mechanisms: 1. Discomfort may arise because the overall amount of activity of the neurons in the network, or activation level, is too high; 2. Discomfort may arise because too many neurons have a high activity, i.e., the encoding is not sparse enough; 3. Discomfort may arise when activity in the hypercolumns is concentrated at a given orientation, i.e., when the isotropy in the network response is low.

With our second experiment, we showed that the same three mechanisms may also shed light into interindividual differences in susceptibility to visual discomfort. To this end, we took advantage of the separation between excitatory and inhibitory populations in neurodynamic models based on Wilson and Cowan formalism (Wilson and Cowan, 1972, 1973), which does not exist in models based on divisive normalization (Heeger, 1992; Foley, 1994; Cavanaugh et al., 2002; Kay et al., 2013; Schutt and Wichmann, 2017), in order to modify the balance between excitation and inhibition. When the balance took higher ratios, e.g., because of a deficit of GABA neurotransmitter availability, the three markers of discomfort shifted toward values associated with increased visual discomfort, namely a higher activation, a lower sparseness, and a decreased isotropy in the model population response. The modelling therefore predicts that more and more stimuli will lead to discomfort as the availability of inhibition lowers. This first mechanistic account for individual difference in susceptibility to visual discomfort corroborate previous findings of a link between GABA concentration and brain activation. There is evidence for variation in GABA levels within the normal population (Cai et al., 2012). Resting GABA concentration predicts peak gamma frequency and fMRI amplitude in response to visual stimulation in humans (Muthukumaraswamy et al., 2009) in response to striped patterns (Adjamian et al., 2004; Muthukumaraswamy et al., 2009). Intriguingly, our modelling makes a link between reduced inhibition and stripes or gratings, visually uncomfortable stimuli par excellence (Wilkins et al., 1984; Hermes et al., 2017): As inhibition was reduced in the model, the isotropy of the model response, which measures the balance of activity across spatial orientations, was progressively lost in a winner-takes-all process in which a single orientation would take all the activity, as in the presence of stripes. Together, our experiments support a unified view of visual discomfort in which core properties of the early visual system – cells sensitive to oriented luminance edges at different spatial orientations and frequencies coupled through a pattern of excitatory and inhibitory connections that implement contextual influences – partially account for both image-wise differences in induced discomfort and individual differences in susceptibility to visual discomfort.

Although a link between visual discomfort and an exceptionally strong neural response has long been proposed (Wilkins et al., 1984), empirical evidence has only emerged recently. Le et al. (2017) used near infrared spectroscopy to measure the oxyhaemoglobin response from posterior areas of the cortex. The amplitude of the response to images of buildings increased with the extent to which the image statistics deviated from $1/f^{\alpha }$. When focusing on colour, striped patterns comprising two colours were more uncomfortable when the chromaticity differences were large (e.g., red and blue) compared to small (pink and purple). The large chromaticity differences also elicited a larger haemodynamic response in visual cortex (Haigh et al., 2013), greater alpha suppression (Haigh et al., 2018), greater steady-state evoked potentials (Lindquist et al., 2021), and larger event-related potentials (ERPs) (Haigh et al., 2019). Individuals with migraine also reported greater discomfort and larger ERPs compared to headache-free individuals (Haigh et al., 2019), further supporting the link between discomfort and strong neural responses.

To our knowledge, the only modelling attempts to understand the neural basis of discomfort so far is (Hibbard and O'Hare, 2015), in which the authors explored a putative relationship between discomfort and sparse coding by computing the kurtosis of the distribution of responses of a set of Gabor units tuned to several spatial frequencies and orientations similar to the first component of our model. The response to natural images was much sparser than that to sine gratings. Our study extends these findings using a model that considers contextual modulations of neurons’ activity outside their CRF, carried out through lateral connections, and to images of real urban scenes and abstract art within a continuum of levels of reported discomfort, instead of the two theoretically extreme corpuses of images formed by natural images and sine gratings. In future works, considering larger, more diverse datasets, for example including interior scenes, would provide insights into the generalizability of our results to other facets of daily life.

A prevailing thesis in vision science is that the evolution of the visual system has been driven by the selective pressure to provide an encoding of natural stimuli that is efficient, both in terms of transmission of information and metabolism (Attneave, 1954; Barlow, 1961). For example, there is strong evidence supporting a sparse representation of natural images in the visual cortex (Vinje and Gallant, 2000, 2002; Weliky et al., 2003; Haider et al., 2010). Efficient coding predicts that natural stimuli are comfortable to view, which is possibly a basis for the restorative effect of nature (Menzel and Reese, 2022). However, and in apparent contradiction with the efficient coding hypothesis, several studies have shown an association between deviation from the statistics of natural images and decreased BOLD response. In (Isherwood et al., 2017), synthetic noise images with $1/f^{\alpha }\;$spectra triggered a stronger BOLD fMRI response in the early visual cortex for a slope closer to 1/f than for steeper or shallower slopes. In (Olman et al., 2004), the BOLD fMRI response to natural images was stronger than to their whitened counterpart. In Rieger et al. (2013), randomising phases in natural images, thereby removing edge information to which the visual system is putatively adapted, had no impact on population metabolic cost as measured by blood oxygenation level dependent (BOLD) response.

The question then is, how to reconcile these findings and the theory of efficient coding? Although the theory predicts that natural stimuli are efficiently processed, it does not follow logically that deviations with respect to nature cause inefficient neural processing. Indeed, efficient coding entails that, considering all possible images, natural images are included in the subset of images processed efficiently, along with some non-natural images. And indeed, this inclusion is strict: non-natural images with a reduced dynamic range of contrast or artificial square-wave gratings with very low frequencies are processed typically. To explore this question computationally, we contrasted the activation of our model against two measures of deviations with respect to 1/f: one that sums deviation in amplitude contrast with respect to natural images at any frequency and spatial orientation in the full two-dimensional amplitude spectrum (Penacchio and Wilkins, 2015), and the slope of the amplitude spectrum (e.g., Tolhurst et al., 1992), as used in Olman et al. (2004) and Isherwood et al. (2017). We found strong correlations between activation and deviation with respect to the full two-dimensional spectrum of natural images (Penacchio and Wilkins, 2015) for all sets but Art 2, but no correlation with the spectral slope (Supplementary Material S9, Table and Figures S16–S19). This computational finding is in agreement with our former empirical measurements in (Le et al., 2017), in which we found an association between increased metabolism and higher two-dimensional deviation with respect to 1/f (Penacchio and Wilkins, 2015). Overall, this suggests that BOLD fMRI responses are more tuned to the amount of contrast at different spatial scales and regions than to the spectral slope (Field and Brady, 1997; Isherwood et al., 2017), a dependence better captured by measuring deviations in the full two-dimensional spectrum (Penacchio and Wilkins, 2015). This calls for a theoretical and empirical characterisation of the deviations from the statistics of natural scenes that lead to inefficient processing.

Nevertheless, several studies have shown that visual discomfort is predicted by the spatial distribution of luminance contrast (Fernandez and Wilkins, 2008; Juricevic et al., 2010; O'Hare and Hibbard, 2011; Penacchio and Wilkins, 2015) or the extent to which the difference in chromaticity (Penacchio et al., 2021) deviates from that found in nature. In our study, the third marker of discomfort, isotropy, shows a similar relationship. In the two sets in which this metric had a large range of values (i.e., urban landscapes), how the isotropy of the encoding on an image differed from the typical values for natural images predicted how uncomfortable to look at it was. The study therefore confirms that discomfort can be triggered by some types of deviation from nature while giving new insights into how image spatial content (or excessive excitation/inhibition ratio) harnesses this deviation.

More generally, by offering three distinct, if partly correlated, mechanisms by which discomfort arises, the study provides a direct and intuitive account for the propensity of simple, laboratory stimuli based on gratings to provoke discomfort, headaches or seizures in photosensitive epilepsy (see Wilkins, 1995; Hermes et al., 2017). The likelihood of causing discomfort increases with the size or the contrast of the gratings (as does the model activation level) and increases if the gratings’ frequency is closer to three cpd (the predictive power of all three markers is higher around this frequency for the experiments in which the visual angle of the stimuli was controlled). This likelihood decreases when superimposing gratings with different orientations (plaid patterns), therefore increasing the isotropy of the model response (Wilkins, 1995).

Nonetheless, the modelling approach is subject to important limitations. As it does not include the machinery of colour processing, the model cannot account for the contribution of chromatic information to discomfort (Haigh et al., 2013; Penacchio et al., 2021; O'Hare et al., 2023). More importantly, while visual stress, migraine, and photosensitive epilepsy have in common a heightening of metabolic and neural response, there is the caveat that our model may not be able to capture the temporal aspect of epilepsy. Epileptogenic stimuli not only trigger a hyperneuronal and hypermetabolic response as in discomfort and migraine (Haigh et al., 2012), but also a temporal synchronization in this response (Binnie et al., 1985). There is strong evidence of an association between seizure generation and oscillations in the gamma band (30–80 Hz) (Hermes et al., 2017). Our firing-rate model is based on Wilson-Cowan equations (Wilson and Cowan, 1972; Wilson and Cowan, 1973). Such equations are not able to generate the fast oscillations needed for monitoring activity in the gamma band (Devalle et al., 2017). Lastly, the neural hyperexcitability underlying differences in susceptibility to visual discomfort may not simply rest on a higher ratio of glutamatergic excitation over GABAergic inhibition, but on more complex interconnections between several types of neurotransmitters (Saxena et al., 2021). A natural progression of this work is to augment the modelling by adopting more elaborate equations that allow the generation of fast oscillations (Devalle et al., 2017) or by using spiking networks, and to consider a bigger diversity of neuron and neurotransmitter types (Shaw et al., 2020).

This study demonstrates the ability for a model of the early visual system to account for two fundamental aspects of visual discomfort, image-wise differences in induced discomfort and individual differences in susceptibility to visual discomfort. Three markers of model activity predicted more than 40 percent of the variance in observers’ reported discomfort from complex, real-life images. More efforts to integrate idiosyncratic differences linked to hyperexcitability in the modelling approach are now central to generate tools that make individual predictions.

## Data availability statement

The datasets presented in this study can be found in online repositories. The names of the repository/repositories (Penacchio et al., 2023) and accession number(s) can be found in the article/Supplementary material.

## Ethics statement

The studies involving human participants were reviewed and approved by Institutional Review Board at the University of Nevada, Reno (333057). The patients/participants provided their written informed consent to participate in this study.

## Author contributions

OP: conceptualization, visualisation, and writing – original draft preparation. OP and XO: data curation, methodology, and software. OP, XO, and AW: formal analysis. OP, XO, SH, and AW: investigation. OP, XO, and SH: Resources. OP, AW, SM, and XO: writing – review and editing. All authors contributed to the article and approved the submitted version.

## Funding

This publication is part of the R+D+I grant PID2020-118254RB-I00 financed by MCIN/AEI/10.13039/501100011033, by the Agencia de Gestió d’Ajuts Universitaris i de Recerca (AGAUR) through 2021-SGR-01470, and the CERCA Programme/Generalitat de Catalunya. OP was funded by a Maria Zambrano Fellowship for attraction of international talent for the requalification of the Spanish university system—NextGeneration EU (ALRC). SH was funded by a pilot award from a NIH COBRE (PG20GM103650) and salary support from a NIH R15 AREA grant (MH122935).

## Conflict of interest

The authors declare that the research was conducted in the absence of any commercial or financial relationships that could be construed as a potential conflict of interest.

## Publisher’s note

All claims expressed in this article are solely those of the authors and do not necessarily represent those of their affiliated organizations, or those of the publisher, the editors and the reviewers. Any product that may be evaluated in this article, or claim that may be made by its manufacturer, is not guaranteed or endorsed by the publisher.
