# Supplementary material for: A mechanistic account of visual discomfort
Source: Front Neurosci. 2023 Jul 20;17:1200661. doi: 10.3389/fnins.2023.1200661 (PMC10397803; doi:10.3389/fnins.2023.1200661)
Supplement: Supplementary file 1 [file Data_Sheet_1.PDF]

Electronic Supplementary Material

Title: **A mechanistic account of visual discomfort**

Authors: **Olivier Penacchio, Xavier Otazu, Arnold J. Wilkins, Sarah M. Haigh**

Supplementary Methods

S1. Model implementation

Component 1. The first component of the model was made of a layer of units reminiscent of cortical simple cells and modelled using Gabor functions. The profile of the Gabor functions were in all identical as the ones presented in (Serre and Riesenhuber 2004, Serre, Oliva et al. 2007). We considered four regularly spaced orientations (0º, 45º, 90º and 135º) and eight 'subpopulations' (frequency channels) of the model corresponding to cells sensitive to different spatial scales with receptive fields of sizes $\mathrm{Rfsize} = [5,7,9,13,17,21,25,31,37,43,49,55]$. Each receptive field included approximatively $\delta = 3$ cycles. The spatial frequencies of the eight spatial scales were, respectively, $\mathrm{ScFr} = [10.8,7.7,6,4.1,3.2,2.6,2.2,1.7,1.5,1.3,1.1,1]$ cycles per degree (cpd) in the viewing conditions of the experiment (see below, Section *Correspondence between frequency channel in the model and visual angle in the experiment*, for a derivation). Please note that it only applied to the sets of stimuli Architecture 1 and 2 and not to Art 1 and 2 as the latter sets were rated online, with no control of the viewing conditions. Each filter was applied at each position in the image. As the sampling was dense, we did not consider different phases for the units. The number of units in each subpopulation sensitive to a given orientation and spatial frequency was the same for the 12 frequency channels. The Gabor filters were normalized so that the sum of their values was 0 and that of the square of their values was 1.

Model 'component 2'. The second component of the model is a firing-rate excitatory-inhibitory network made of a population of excitatory cells with membrane potentials $(x_{is\theta})$ and inhibitory cells with membrane potentials $(y_{is\theta})$ organized into a regular grid of hypercolumns of size 256 x 256, *i.e.*, one hypercolumn for each pixel in the input images, where each excitatory or inhibitory unit is characterised by a triple $[i, s, \theta]$, with i being the location of the hypercolumn it belongs to and the centre of the receptive field of the unit, s refers to one of the eight subpopulations of the model sensitive to different spatial frequencies, and θ is the preferred orientation of the unit. Pairs of excitatory units in the network, $x_{is\theta}$ and $x_{js'\theta'}$, are connected through lateral connections of strength $J_{[is\theta,js'\theta']}$ set up to enhance 'collinear activation' of roughly aligned features, namely to boost the mutual reinforcement of the activity of cells whose respective locations and relative orientations may respond to a typical contour in natural scenes (Knierim and Vanessen 1992, Kapadia, Ito et al. 1995, Weliky, Kandler et al. 1995). Pairs of inhibitory and excitatory units, $y_{is\theta}$ and

$x_{js'\theta'}$, are connected through lateral connections of strength $W_{[is\theta,js'\theta']}$ set up to mutually inhibit the activity of cells sensitive to edges that are roughly parallel through disynaptic connections (see (Li 1999, Penacchio, Otazu et al. 2013) for a schematic of the patterns of connections J and W). The firing rates of the excitatory and inhibitory units are given by the output of non-linear monotonic increasing activation functions $x_{is\theta} \rightarrow g_x(x_{is\theta})$ and $y_{is\theta} \rightarrow g_y(y_{is\theta})$, respectively.

The dynamic of the network is driven by the following differential equations

$$\begin{cases} \dfrac{dx_{is\theta}}{dt} = -\alpha_x x_{is\theta} - g_y(y_{is\theta}) - \displaystyle\sum_{\Delta s, \Delta\theta \neq 0} \psi(\Delta s, \Delta\theta) g_y(y_{is+\Delta s\theta+\Delta\theta}) + J_0 g_x(x_{is\theta}) \\ \qquad + \displaystyle\sum_{j \neq i, s', \theta'} J_{[is\theta, js'\theta']} g_x(x_{js'\theta'}) + I_{is\theta} + I_0, \\ \dfrac{dy_{is\theta}}{dt} = -\alpha_y y_{is\theta} + g_x(x_{is\theta}) + \displaystyle\sum_{j \neq i, s', \theta'} W_{[is\theta, js'\theta']} g_x(x_{js'\theta'}) + I_c, \end{cases}$$

where

- α_x and α_y are constant that control the temporal reactivity of the network;
- ψ is a function that implements inhibition between cells sensitive to similar orientations within each hypercolumn;
- J_0 models self-excitatory activity;
- I_0 is a normalization term;
- I_c describes the background input to the inhibitory layer;
- $I_{is\theta}$ is the (constant for each image processed) visual input to the network given by the output of the units that make 'component 1'.

The values of the parameters of the network have not been fitted for this work and are in all identical to those described in previous works (see (Li 1999), and (Penacchio, Otazu et al. 2013), Supporting Information, for a full description of all the parameters).

Modification of the excitation/inhibition balance. The ratio of excitation to inhibition in the model was first manipulated by modifying the activation functions of the inhibitory layer of the model $y \rightarrow g_y(y)$ using a multiplicative gain γ, as

$$y \rightarrow \gamma g_y(y).$$

The gain varied between 0 (no inhibition at all in the network) to 1 (the reference model).

Correspondence between frequency channel in the model and visual angle in the experiment.

(Please note that this correspondence only applies for the stimuli rated in the laboratory, i.e., for the

results relative to the sets Architecture 1 and 2). The peak frequency, in cycles per image, for a receptive field of size RFsize consisting of δ cycles, is

$$N\delta/Rfsize$$

for a square image of size N pixels. The peak frequency of a receptive field in the experimental conditions, in cycles per degree (cpd), is therefore

$$N\delta/(Rfsize\theta),$$

where θ is the visual angle of the image in the experiment. The peak frequencies of the units in the model were therefore $\mathrm{ScFr} = [10.8,7.7,6,4.1,3.2,2.6,2.2,1.7,1.5,1.3,1.1,1]$, hence ranging between 1 cpd (biggest receptive fields, size 55x55 pixels) and 10.8 cpd (smallest receptive fields, size 5x5 pixels), with an average of 3.5 cpd, in fair agreement with electrophysiological recordings (*e.g.*, 0.5 to 8 cpd, average 2.2 cpd in (Devalois, Albrecht et al. 1982)). Note that the spatial frequencies we considered were not exactly logarithmically spaced. A proper logarithmic spacing with the same number of channels (12) and with spatial frequencies ranging between 1 cpd and 10.8 cpd would have led to spatial frequencies of [1.0000, 1.24, 1.54, 1.91, 2.38, 2.95, 3.66, 4.55, 5.64, 7.00, 8.70, 10.8], which would have provided a spatial frequency sampling very similar to the one chosen.

S2. Non-classical receptive field stimulation increases the sparseness of the model response

The excitatory-inhibitory neurodynamical model used in this work has been shown to reproduce several phenomena that take place, at least in part, in the early visual cortex, namely figure-ground segmentation, contour grouping and bottom-up saliency (Zhaoping and May 2007, Zhang, Zhaoping et al. 2012, Zhaoping and Zhe 2015, Berga and Otazu 2020, Berga and Otazu 2022), with a good fit with behavioural experiment and neuroimaging data (Zhang, Zhaoping et al. 2012, Zhaoping and Zhe 2015), and, qualitatively, brightness induction (Penacchio, Otazu et al. 2013).

Stimulating simultaneously the classical receptive field (CRF) and the nonclassical receptive field (nCRF) of a cortical visual neuron with naturalistic stimuli increases the sparseness of the neuron response (Vinje and Gallant 2000, Haider, Krause et al. 2010). To test whether the stimulation of regions contiguous to the CRF had an influence on the sparseness of the response of the model, we analysed how the sparseness of the activity of a central set of units evolved when the stimulation area was increased from a small region to a wide region. The restriction of the stimulation area was done by applying to all the images in Set 4 circular masks with different radii and centred at the same location (corresponding to a reference hypercolumn located at the centre of the image) (Vinje and Gallant 2000). To reduce border effects, the masks were smoothed beforehand using a Gaussian kernel (σ =1/2 pixel). The effect of the mask on an image was to set to zero all the pixel values

outside of the circular area within which the mask had positive values. The radius of the circular stimulation area ranged from 3.5 pixels (corresponding approximatively to the size of the smaller CRF, namely those of the units sensitive to the highest spatial frequencies) to 53.5 pixels in steps of 2 (3.5 to 17.5) then 4 (17.5 to 53.5), resulting in 17 different masks. The activity of the model was therefore computed for 74 (number of images in Set 4) x 17 (number of masks) = 1258 different input images.

For each of the 4 (orientations in each hypercolumn) x 8 (scales in each hypercolumns) = 32 units in the central hypercolumn and for each radius size, we obtained a distribution of response activity by concatenating the firing rates for the 74 images and the membrane time constants once the steady state was reached (*i.e.*, between the 5th and the 20th membrane time constants, see main text). We next analysed the lifetime sparseness of all the 32 units separately by computing the kurtosis of the corresponding distribution of firing rates for each unit (figure S1a). We also analysed the population sparseness of the population made by all the units in the reference hypercolumn by concatenating all the distributions of firing rates of the individual units (figure S1b).

Figure S1a shows that the median lifetime sparseness of the individual responses of the units strongly increased when extending the area of visual stimulation. Increasing the diameter of the stimulation from the smallest value (radius = 3.5) to twice this value (radius = 7.5), two and a half times this value (radius = 9.5) and four times this value (radius = 15.5) resulted in significantly different kurtosis distributions (Kolmogorov-Smirnov test: D(radius = 3.5, radius = 7.5) = 0.27, $p < 0.05$; D(radius = 3.5, radius = 9.5) = 0.437, $p < 10^{-4}$; D(radius = 3.5, radius = 15.5) = 0.604, $p < 10^{-7}$). There was no significant increase in lifetime sparseness when the stimulation region was further enlarged (*e.g.*, the difference between stimulating an area of radius 17.5 and stimulating an area of radius 53.5 was not significant, Kolmogorov-Smirnov statistic D(radius = 17.5, radius = 53.5) = 0.187, $p = 0.33$; none of the differences between pairs of distributions for a radius beyond 17.5 was significant, all $p > 0.139$).

Figure S1b shows that the sparseness of the whole population also increased dramatically when increasing the radius of the stimulation until the stimulation area reached the size of 5-6 receptive fields of the units tuned to the highest spatial frequency. Taken together, these results show that the model replicates the findings that stimulating the nCRF of a cortical visual neuron in addition to its CRF with naturalistic stimuli increases the sparseness of its response (Vinje and Gallant 2000, Haider, Krause et al. 2010).

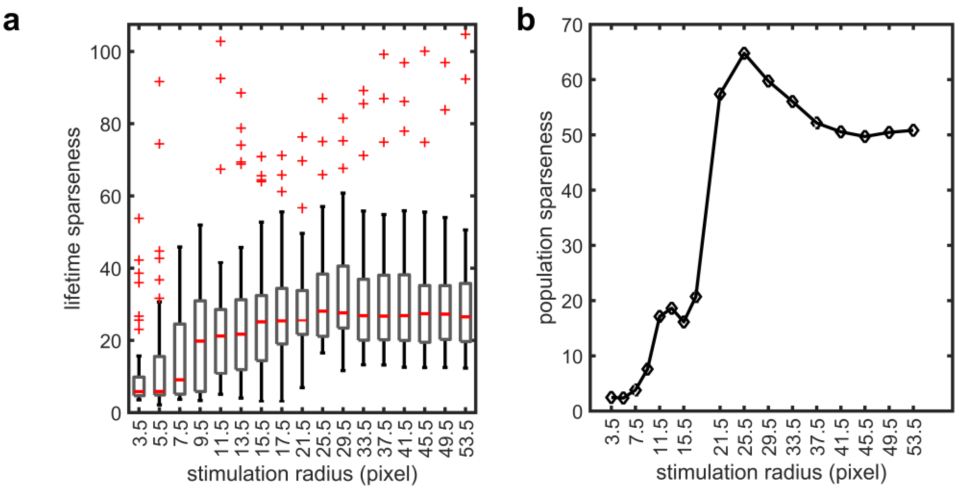

Figure S1. Increasing the area of stimulation increases lifetime sparseness of individual units and population sparseness. (a) Distributions of lifetime sparseness for 32 units (4 orientations, 8 spatial frequencies) located at the centre of the retinotopic grid in function of the radius of the stimulation area. The notch boxes show 95% confidence interval of the median, the interquartile range (IQR) and the lower (resp. upper) whisker show the 25 percentile (resp. 75 percentile) minus 1.5 IQR (resp. plus 1.5 IQR). (b) Sparseness of the whole population (the 32 central units considered together) as a function of the radius of the stimulation area.

S3. Alternative metrics

We assessed alternative measures for two of the three types of makers, namely activation and sparseness.

For activation, we also measured the $L^{0.5}$, $L^{1.5}$, L^{2}, $L^{2.7}$-norm of the model population response as $\|(x_{is\theta}(t))\|_p = (\sum_{i,\theta,s,t} |x_{is\theta}(t)|^p)^{1/p}$, with $p\ =\ 0.5, 1.5, 2$ and 2.7 and the standard deviation of the model population response. We found strong correlations between these alternative measures of activation and the measure used in the text (L^1), and very similar correlations with observers' ratings of discomfort (see Figures S2-S5 below).

For sparseness, we also measured the kurtosis of the model population response (Hibbard and O'Hare 2015), its Gini index (Hurley and Rickard 2009), and the rate parameter obtained when fitting exponential distributions to the distribution of firing rates (“exponential decay”, see (Baddeley, Abbott et al. 1997)). We found relatively good correlations between most of these measures, with relatively similar prediction of observers' ratings of discomfort for the set Architecture 1 and 2, apart from the measure provided by the Gini index (see Figure S2-S5 below).

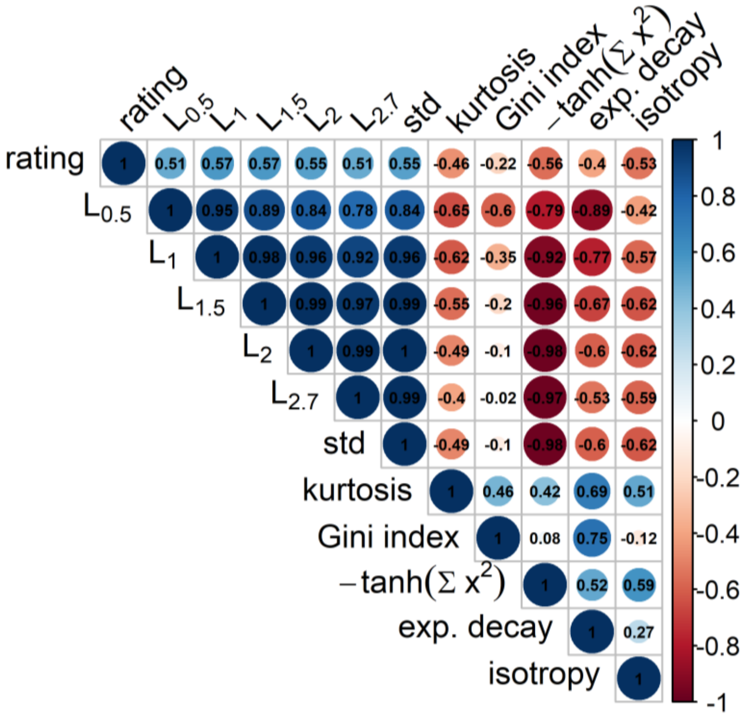

Figure S2. Correlation between the three markers of discomfort used in the manuscript (activation, "L^1"; sparseness "$-\sum \tanh x^2$"; isotropy, "isotropy") and the alternative measures for set Architecture 1 (N=75). Colours provide the Pearson correlation coefficient between two measures, or a measures and observers' average reported discomfort, with dark blue corresponding to a perfect correlation and dark red to a perfect anticorrelation. Blank entries correspond to non-significant correlations at the 0.05 level.

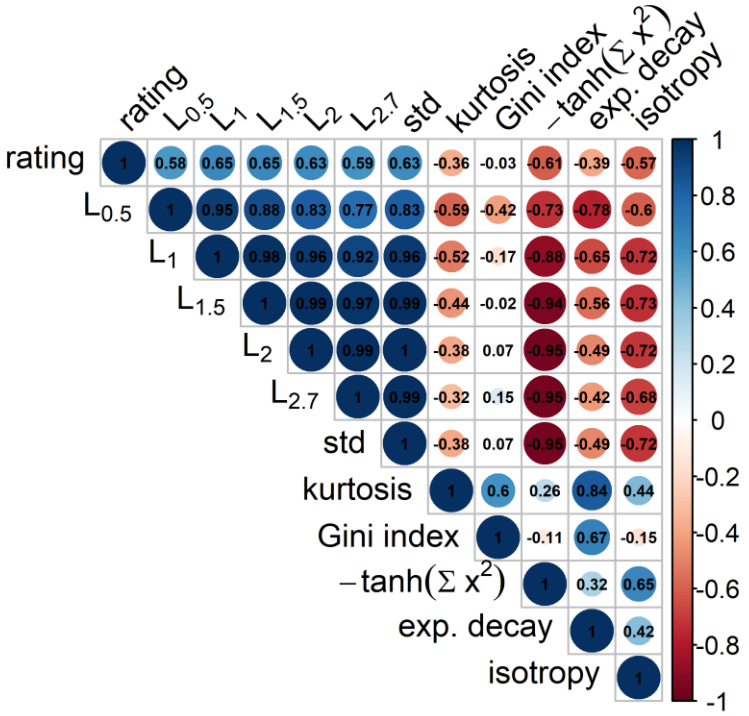

Figure S3. Correlation between the three markers of discomfort used in the manuscript (activation, "L^1"; sparseness "$-\sum \tanh x^2$"; isotropy, "isotropy") and the alternative measures for set Architecture 2 (N=75). All conventions as in Figure S2.

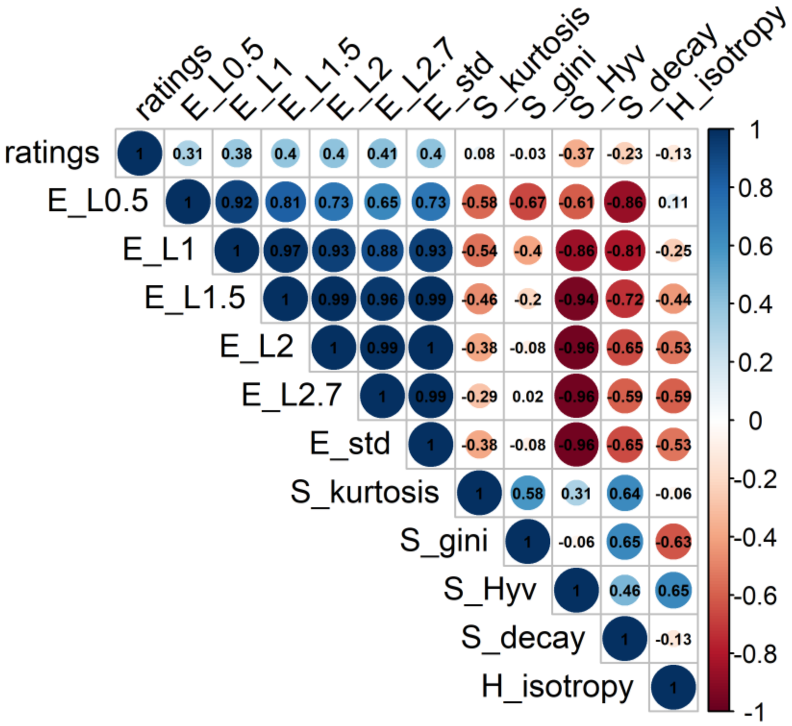

Figure S4. Correlation between the three markers of discomfort used in the manuscript (activation, "$\mathbf{L^1}$"; sparseness "$-\sum \tanh x^2$"; isotropy, "isotropy") and the alternative measures for set Art 1 (N=50). All conventions as in Figure S2.

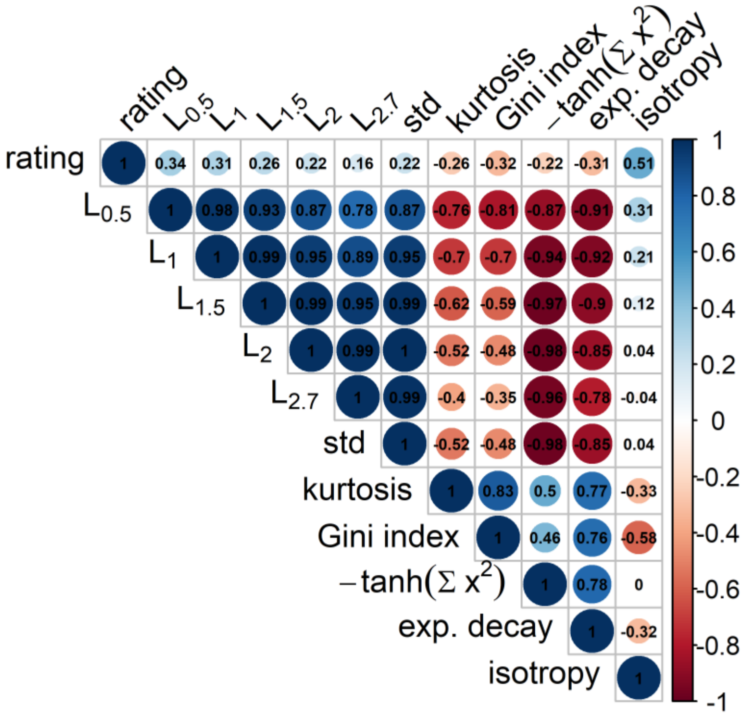

Figure S5. Correlation between the three markers of discomfort used in the manuscript (activation, "$\mathbf{L^1}$"; sparseness "$-\sum \tanh x^2$"; isotropy, “$\mathbf{isotropy}$”) and the alternative measures for set Art 2 (N=50). All conventions as in Figure S2.

Supplementary results

S4. Statistical inference

Experiment 1

Models used in the inference process for Experiment 1. In each case, the model chosen is highlighted in grey.

▶ **POPULATION ACTIVITY LEVEL (E) regressed against reported discomfort in set:**

ARCHITECTURE 1

Model	Nested Model	Effects		AIC	BIC	Log Likelihood	Likelihood Ratio test		
		Fixed	Random over subject (experimental setting)				df	χ^2	p-value
**modNull**			Intercept	2703	2717	-1349			
**modE**	modNull	+ E		2645	2664	-1319	1	60.23	$< 10^{-14}$
**modErs**	modE		+ E	2602	2630	-1295	2	46.93	$< 10^{-10}$

Model selected modErs: rating ~ E + (E|subject)

**Fixed effects**	Estimate	SE	t-value
**Intercept**	3.71	0.23	16.28
**E**	0.84	0.29	2.90

**Random effects**	Variance	SD
**Subject**	0.49	0.70
**E**	0.74	0.86

Model fit: R^2 (marginal) 0.066; R^2 (conditional) 0.321

ARCHITECTURE 2

Model	Nested Model	Effects		AIC	BIC	Log Likelihood	Likelihood Ratio test		
		Fixed	Random over subject (experimental setting)				df	χ^2	p-value
**modNull**			Intercept	2799	2812	-1396			
**modE**	modNull	+ E		2664	2682	-1328	1	136.61	$< 10^{-15}$
**modErs**	modE		+ E	2647	2675	-1318	2	20.79	$< 10^{-4}$

Model selected modErs: rating ~ E + (E|subject)

**Fixed effects**	Estimate	SE	t-value
**Intercept**	3.39	0.15	22.58
**E**	1.30	0.22	6.04

**Random effects**	Variance	SD

**Subject**	0.20	0.45
**E**	0.36	0.60

Model fit: R^2 (marginal) 0.159; R^2 (conditional) 0.266

ART 1

Model	Nested Model	Effects		AIC	BIC	Log Likelihood	Likelihood Ratio test		
		Fixed	Random over subject (experimental setting)				df	χ^2	p-value
**modNull**			Intercept	6359	6377	-3177			
**modE**	modNull	+ E		6327	6350	-3159	1	34.32	$< 10^{-8}$
**modErs**	modE		+ E	6266	6302	-3127	2	64.40	$< 10^{-13}$

Model selected modErs: rating ~ E + (E|subject)

**Fixed effects**			
	**Estimate**	**SE**	**t-value**
**Intercept**	1.59	0.7	22.32
**E**	0.19	0.05	3.54

**Random effects**		
	**Variance**	**SD**
**Subject**	0.26	0.51
**E**	0.11	0.33

Model fit: R^2 (marginal) 0.010; R^2 (conditional) 0.340

ART 2

Model	Nested Model	Effects		AIC	BIC	Log Likelihood	Likelihood Ratio test		
		Fixed	Random over subject (experimental setting)				df	χ^2	p-value
**modNull**			Intercept	9897	9916	-4945			
**modE**	modNull	+ E		9846	9871	-4919	1	52.60	$< 10^{-12}$
**modErs**	modE		+ E	9824	9862	-4906	2	25.68	$< 10^{-5}$

Model selected modErs: rating ~ E + (E|subject)

**Fixed effects**			
	**Estimate**	**SE**	**t-value**
**Intercept**	1.72	0.06	26.78
**E**	0.20	0.04	5.77

**Random effects**		
	**Variance**	**SD**
**Subject**	0.31	0.56
**E**	0.04	0.20

Model fit: R^2 (marginal) 0.010; R^2 (conditional) 0.337

▶ **SPARSENESS OF MODEL RESPONSE (S) regressed against reported discomfort in set:**

ARCHITECTURE 1

Model	Nested Model	Effects		AIC	BIC	Log Likelihood	Likelihood Ratio test		
		Fixed	Random over subject (experimental setting)				df	χ^2	p-value
**modNull**			Intercept	2703	2717	-1349			
**modS**	modNull	+ S		2649	2668	-1321	1	56.05	$< 10^{-13}$
**modSrs**	modS		+ S	2637	2665	-1313	2	16.11	$< 10^{-4}$

Model selected modSrs: rating ~ S + (S|subject)

**Fixed effects**			
	**Estimate**	**SE**	**t-value**
**Intercept**	3.71	0.23	16.28
**S**	-0.81	0.21	-3.87

**Random effects**		
	**Variance**	**SD**
**Subject**	0.49	0.70
**S**	0.34	1.38

Model fit: R^2 (marginal) 0.062; R^2 (conditional) 0.279

ARCHITECTURE 2

Model	Nested Model	Effects		AIC	BIC	Log Likelihood	Likelihood Ratio test		
		Fixed	Random over subject (experimental setting)				df	χ^2	p-value
**modNull**			Intercept	2799	2812	-1396			
**modS**	modNull	+ S		2682	2700	-1337	1	118.93	$< 10^{-15}$
**modSrs**	modS		+ S	2671	2698	-1329	2	14.90	$< 10^{-4}$

Model selected modSrs: rating ~ S + (S|subject)

**Fixed effects**			
	**Estimate**	**SE**	**t-value**
**Intercept**	3.39	0.15	22.58
**S**	-1.23	0.19	-6.54

**Random effects**		
	**Variance**	**SD**
**Subject**	0.20	0.44
**S**	0.24	0.49

Model fit: R^2 (marginal) 0.140; R^2 (conditional) 0.236

ART 1

Model	Nested Model	Effects	AIC	BIC	Log Likelihood	Likelihood Ratio test

Model	Nested Model	Effects: Fixed	Effects: Random over subject (experimental setting)	AIC	BIC	Log Likelihood	Likelihood Ratio test: df	χ^2	p-value
**modNull**			Intercept	6359	6377	-3177			
**modS**	modNull	+ S		6329	6353	-3161	1	31.90	$< 10^{-7}$
**modSrs**	modS		+ S	6288	6323	-3138	2	45.16	$< 10^{-9}$

Model selected modSrs: rating ~ S + (S|subject)

**Fixed effects**	**Estimate**	**SE**	**t-value**
**Intercept**	1.59	0.07	22.32
**S**	-0.18	0.05	-3.67

**Random effects**	**Variance**	**SD**
**Subject**	0.26	0.51
**S**	0.09	0.29

Model fit: R^2 (marginal) 0.010; R^2 (conditional) 0.333

ART 2

**Model**	**Nested Model**	**Effects**		**AIC**	**BIC**	**Log Likelihood**	**Likelihood Ratio test**		
		Fixed	Random over subject (experimental setting)				df	χ^2	p-value
**modNull**			Intercept	9897	9916	-4945			
**modS**	modNull	+ S		9874	9899	-4933	1	24.74	$< 10^{-6}$
**modSrs**	modS		+ S	9860	9898	-4924	2	17.92	$< 10^{-4}$

Model selected modSrs: rating ~ S + (S|subject)

**Fixed effects**	**Estimate**	**SE**	**t-value**
**Intercept**	1.72	0.06	26.78
**S**	-0.14	0.03	-4.38

**Random effects**	**Variance**	**SD**
**Subject**	0.31	0.56
**S**	0.03	0.18

Model fit: R^2 (marginal) 0.005; R^2 (conditional) 0.330

▶ ANISOTROPY OF MODEL RESPONSE (H) regressed against reported discomfort in set:
ARCHITECTURE 1

**Model**	**Nested Model**	**Effects**		**AIC**	**BIC**	**Log Likelihood**	**Likelihood Ratio test**		
		Fixed	Random over subject				df	χ^2	p-value

Model	Nested Model	Effects: Fixed	Effects: Random over subject (experimental setting)	AIC	BIC	Log Likelihood	Likelihood Ratio test: df	χ^2	p-value
**modNull**			Intercept	2703	2717	-1349			
**modH**	modNull	+ S		2654	2672	-1323	1	51.33	$< 10^{-12}$
**modHrs**	modH		+ S	2637	2665	-1313	2	20.87	$< 10^{-4}$

Model selected modHrs: rating ~ H + (H|subject)

**Fixed effects**	Estimate	SE	t-value
**Intercept**	3.71	0.23	16.28
**H**	-0.78	0.23	-3.45

**Random effects**	Variance	SD
**Subject**	0.49	0.70
**H**	0.41	0.64

Model fit: R^2 (marginal) 0.057; R^2 (conditional) 0.281

ARCHITECTURE 2

Model	Nested Model	Effects: Fixed	Effects: Random over subject (experimental setting)	AIC	BIC	Log Likelihood	Likelihood Ratio test: df	χ^2	p-value
**modNull**			Intercept	2799	2812	-1396			
**modH**	modNull	+ H		2697	2715	-1344	1	103.93	$< 10^{-15}$
**modHrs**	modH		+ H	2683	2710	-1335	2	17.83	$< 10^{-4}$

Model selected modHrs: rating ~ H + (H|subject)

**Fixed effects**	Estimate	SE	t-value
**Intercept**	3.39	0.15	22.58
**S**	-1.15	0.21	-5.39

**Random effects**	Variance	SD
**Subject**	0.20	0.44
**S**	0.35	0.59

Model fit: R^2 (marginal) 0.124; R^2 (conditional) 0.229

ART 1

Model	Nested Model	Effects: Fixed	Effects: Random over subject (experimental setting)	AIC	BIC	Log Likelihood	Likelihood Ratio test: df	χ^2	p-value
**modNull**			Intercept	6359	6377	-3177			
**modH**	modNull	+ H		6362	6385	-3177	1	0.75	0.39

Model selected modNull: the level of anisotropy of the model response did not predict visual discomfort.

ART 2

**Model**	**Nested Model**	**Effects**		**AIC**	**BIC**	**Log Likelihood**	**Likelihood Ratio test**		
		Fixed	Random over subject (experimental setting)				df	χ^2	p-value
**modNull**			Intercept	9897	9916	-4945			
**modH**	modNull	+ H		9746	9771	-4869	1	152.44	$< 10^{-15}$
**modHrs**	modH		+ H	9734	9772	-4861	2	16.41	$< 10^{-4}$

Model selected modHrs: rating ~ H + (H|subject)

**Fixed effects**	**Estimate**	**SE**	**t-value**
**Intercept**	1.72	0.06	26.78
**H**	0.33	0.03	10.51

**Random effects**	**Variance**	**SD**
**Subject**	0.31	0.56
**H**	0.02	0.16

Model fit: R^2 (marginal) 0.027; R^2 (conditional) 0.350

S5. Correlations between metrics

S5.1. *Raw correlations and scatterplots*. The three metrics, model activation level (E), sparseness of the model response (S), and isotropy in the model response (H), were linearly correlated. The Pearson correlation coefficients were r_{ES} (activation vs. sparseness) = -0.92 ($p < 10^{-15}$; ci = [-0.95, -0.88]), r_{EH} (activation vs. isotropy) = -0.57 ($p < 10^{-7}$; ci = [-0.71, -0.40]), r_{SH} (sparseness vs. isotropy) = 0.59 ($p < 10^{-7}$; ci = [0.42, 0.72]) for Architecture 1, r_{ES} = -0.88 ($p < 10^{-15}$; ci = [-0.93, -0.82]), r_{EH} = -0.72 ($p < 10^{-12}$; ci = [-0.82, -0.59]), r_{SH} = 0.65 ($p < 10^{-9}$; ci = [0.50, 0.77]) for Architecture 2, r_{ES} = -0.86 ($p < 10^{-14}$; ci = [-0.92, -0.76]), r_{EH} = -0.25 (n.s., p = 0.08; ci = [-0.49, 0.03]), r_{SH} = 0.65 ($p < 10^{-6}$; ci = [0.45, 0.78]) for Art 1, and r_{ES} = -0.94 ($p < 10^{-15}$; ci = [-0.97, -0.90]), r_{EH} = 0.21 (n.s., p = 0.14; ci = [-0.07, 0.46]), r_{SH} = 0.00 (n.s., p = 0.99; ci = [-0.28, 0.28]) for Art 2. Figure SN-SP below show the relationship between the metrics for the four sets of images.

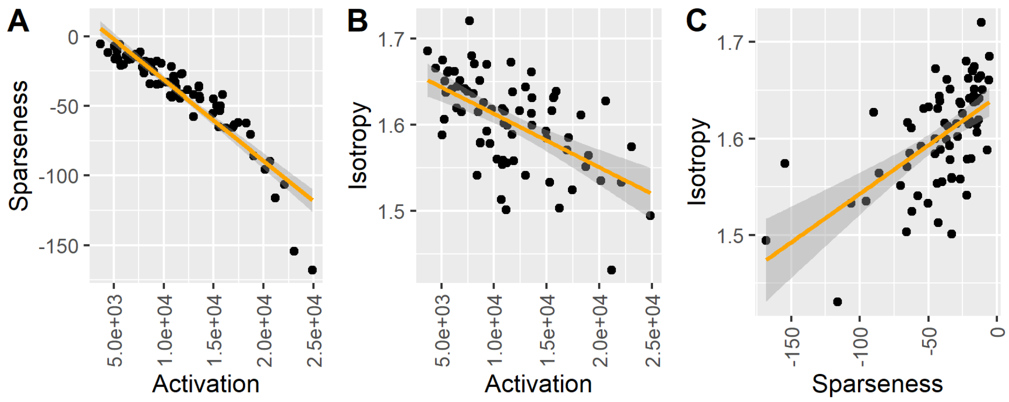

Figure S6. Plots of the three metrics, activation, sparseness, and isotropy against each other for the images in Architecture 1.

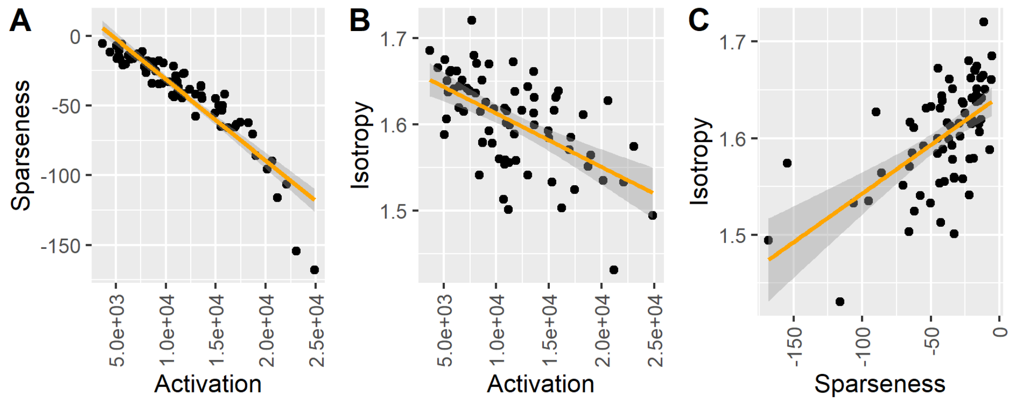

Figure S7. Plots of the three metrics, activation, sparseness, and isotropy against each other for the images in Architecture 2.

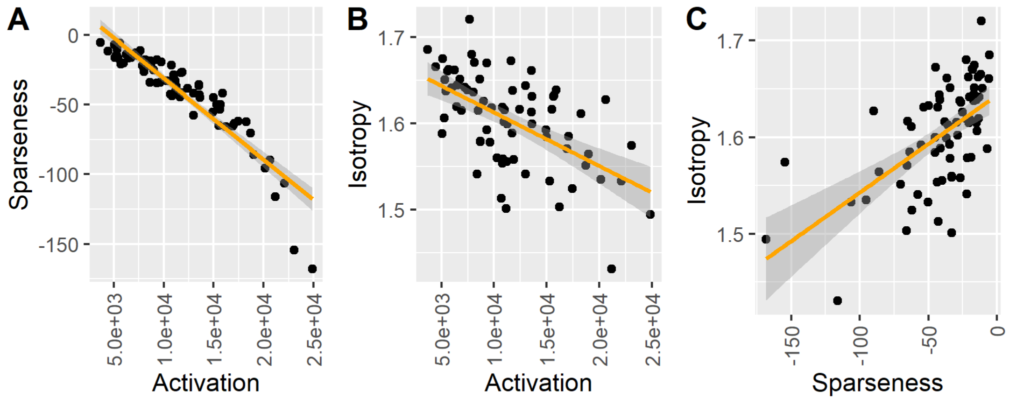

Figure S8. Plots of the three metrics, activation, sparseness, and isotropy against each other for the images in Art 1.

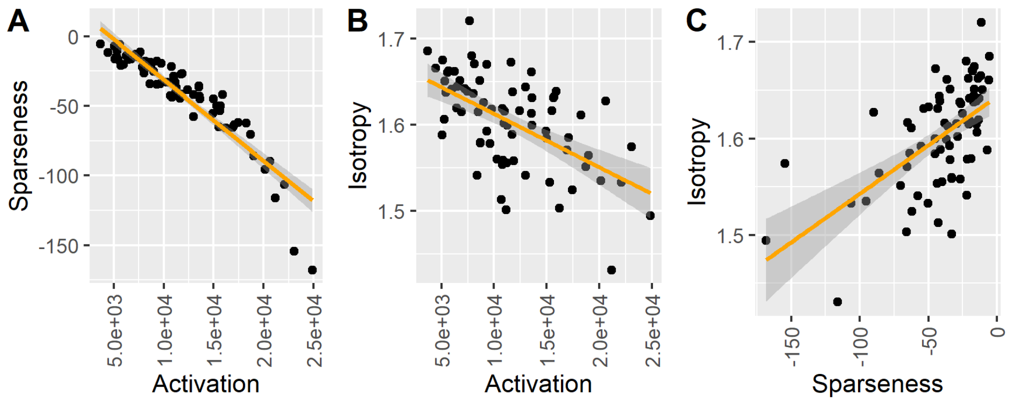

Figure S9. Plots of the three metrics, activation, sparseness, and isotropy against each other for the images in Art 2.

S5.2. *Prediction with all metrics versus a single metric*. Considering the correlations between the metrics, we compared models including the three metrics E, S and H as predictors with models only containing one metric (counterevidence for using a more complex model highlighted in grey):

**Architecture 1**	χ^2	p	ΔAIC	ΔBIC
modErs vs. modESHrs	41.13	$< 10^{-5}$	-23	18
modSrs vs. modESHrs	76.13	$< 10^{-12}$	-58	-17
modHrs vs. modESHrs	76.09	$< 10^{-12}$	-58	-17

**Architecture 2**	χ^2	p	ΔAIC	ΔBIC
modErs vs. modESHrs	20.99	0.013	-3	38
modSrs vs. modESHrs	44.56	$< 10^{-5}$	-27	15
modHrs vs. modESHrs	56.63	$< 10^{-8}$	-39	3

**Art 1**	χ^2	p	ΔAIC	ΔBIC
modErs vs. modESHrs	7.64	0.57	10	63
modSrs vs. modESHrs	29.29	0.00058	-11	42
modHrs vs. modESHrs	103.78	$< 10^{-15}$	-86	-33

**Art 2**	χ^2	p	ΔAIC	ΔBIC
modErs vs. modESHrs	151.89	$< 10^{-15}$	-134	-77
modSrs vs. modESHrs	187.5	$< 10^{-15}$	-169	-113
modHrs vs. modESHrs	61.32	$< 10^{-9}$	-43	13

For Art 1 and 2 we reproduced this analysis for the metrics computed from the activity of the frequency channel that gave the best correlation with observers' ratings:

Art 1	χ^2	p	ΔAIC	ΔBIC
modErs vs. modESHrs	46.18	$< 10^{-6}$	-28	25
modSrs vs. modESHrs	95.59	$< 10^{-15}$	-78	-25
modHrs vs. modESHrs	220.36	$< 10^{-15}$	-202	-149

Art 2	χ^2	p	ΔAIC	ΔBIC
modErs vs. modESHrs	189.49	$< 10^{-15}$	-171	-115
modSrs vs. modESHrs	232.54	$< 10^{-15}$	-215	-158
modHrs vs. modESHrs	219.75	$< 10^{-15}$	-202	-145

S5.3. *Relationship between activation and sparseness*. Given the high correlation between 'activation' and 'sparseness' we compared models including these two predictors to models containing only one (same convention as in the tables above):

Architecture 1	χ^2	p	ΔAIC	ΔBIC
modErs vs. modESrs	23.17	0.00012	-15	3
modSrs vs. modESrs	58.17	$< 10^{-11}$	-50	-32

Architecture 2	χ^2	p	ΔAIC	ΔBIC
modErs vs. modESrs	9.24	0.055	-1	17
modSrs vs. modESrs	32.81	$< 10^{-5}$	-25	-6

Art 1	χ^2	p	ΔAIC	ΔBIC
modErs vs. modESrs	4.86	0.30	3	27
modSrs vs. modESrs	26.52	$< 10^{-4}$	-19	5

Art 2	χ^2	p	ΔAIC	ΔBIC
modErs vs. modESrs	24.14	$< 10^{-4}$	-16	9
modSrs vs. modESrs	59.75	$< 10^{-11}$	-52	-27

We also wondered whether it is possible to find population activities for which activation and sparseness are disentangled. To this end we considered distributions of firing rates modelled using log-normal distributions. (Log-normal distributions fit well populations of firing rates (Linden and Berg 2021).) We considered a single population created by joining two subpopulations drawn from two log-normal distributions with different parameters. By varying the parameters, we found that it was possible to find whole distributions of firing rates with the same level of activation and very different levels of sparseness, as shown in Figure S10 below. It is unlikely that our model or actual neural codes can reach this level of independence between activation and sparseness, but the tables above show that for two sets of images, Architecture 1 and Art 2, activation and sparseness had some degree of independence and considering both metrics did increase the amount of explained variance in discomfort.

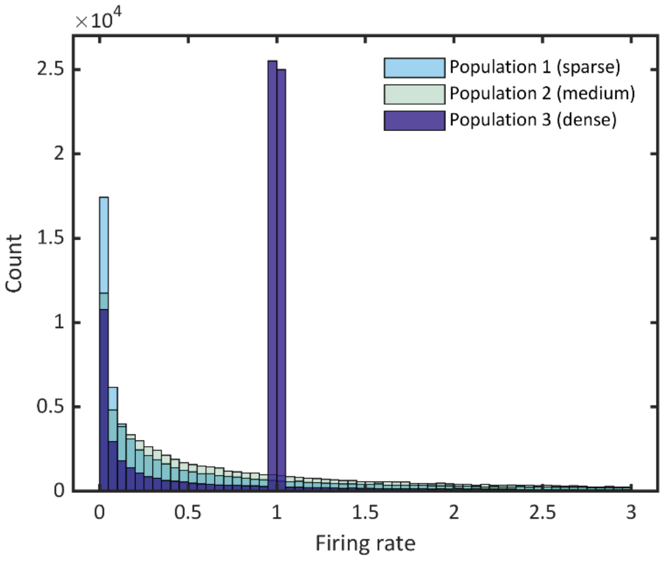

Figure S10. Three population of firing rate with the same level of activation, but three different levels of sparseness.

S6. Impact of excitation/inhibition balance on the three markers for other sets of stimuli

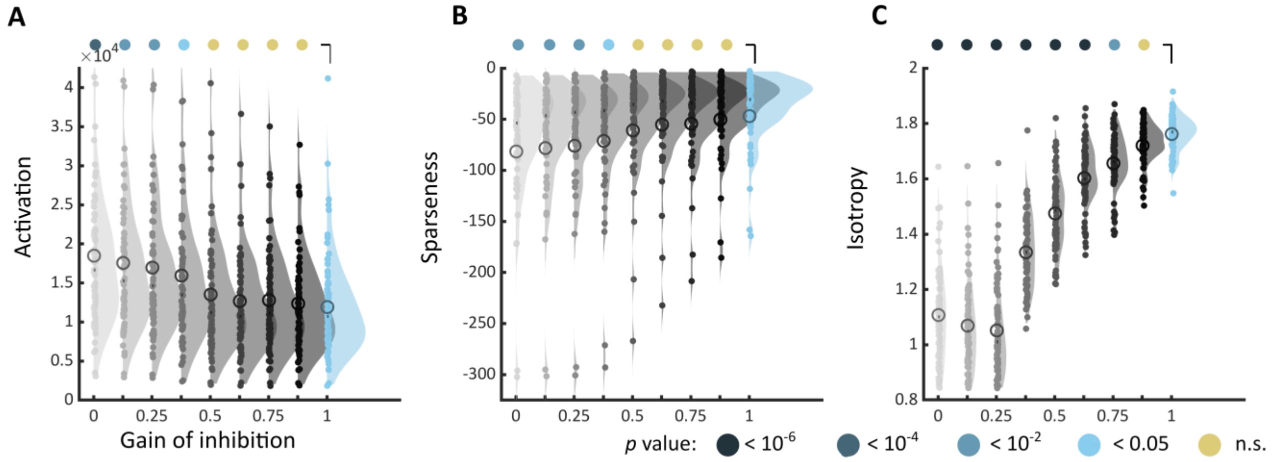

Figure S11. (Counterpart of Figure 5 in the main text for Architecture 2) Changes in markers of visual discomfort when the balance of excitation over inhibition is modified. Distributions of (A) activation, (B) sparseness, and (C) isotropy metrics for all the stimuli in Architecture 2 and increasing values of gain for the inhibitory layer. The gain ranged from 0, i.e., no inhibitory activity in the model (top left, light grey distribution), to 1, *i.e.*, reference model (top right, blue distribution), in steps of 0.125. Differences between distributions and the distribution for the reference model were tested using two-sample Kolmogorov-Smirnov tests; p-values are colour coded as in Figure 3 in the main manuscript.

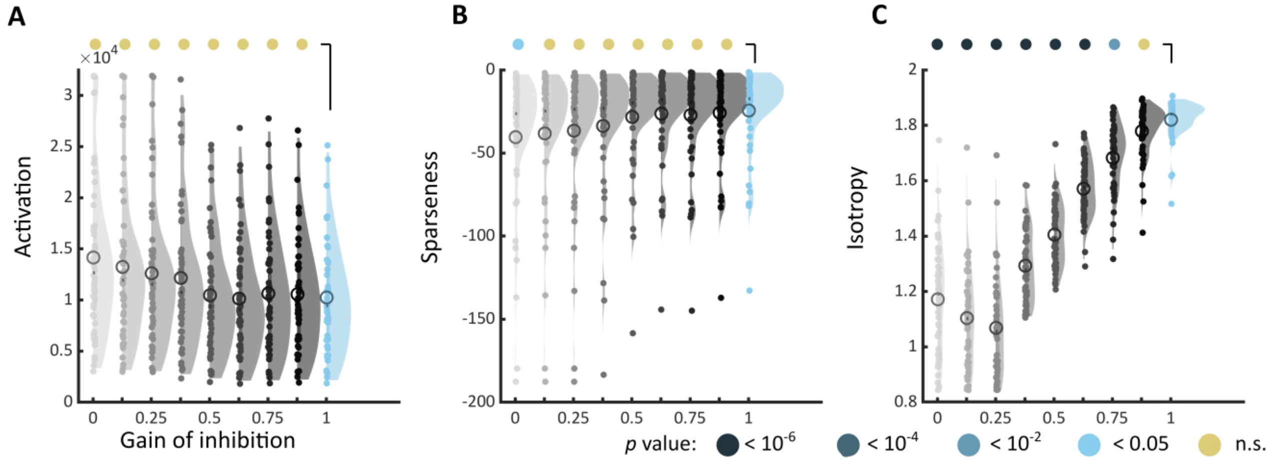

Figure S12. (Counterpart of Figure 5 in the main text for Art 1) Changes in markers of visual discomfort when the balance of excitation over inhibition is modified. Distributions of (A) activation, (B) sparseness, and (C) isotropy metrics for all the stimuli in Art 1 and increasing values of gain for the inhibitory layer. The gain ranged from 0, i.e., no inhibitory activity in the model (top left, light grey distribution), to 1, *i.e.*, reference model (top right, blue distribution), in steps of 0.125. Differences between distributions and the distribution for the reference model were tested using two-sample Kolmogorov-Smirnov tests; p-values are colour coded as in Figure 3 in the main manuscript.

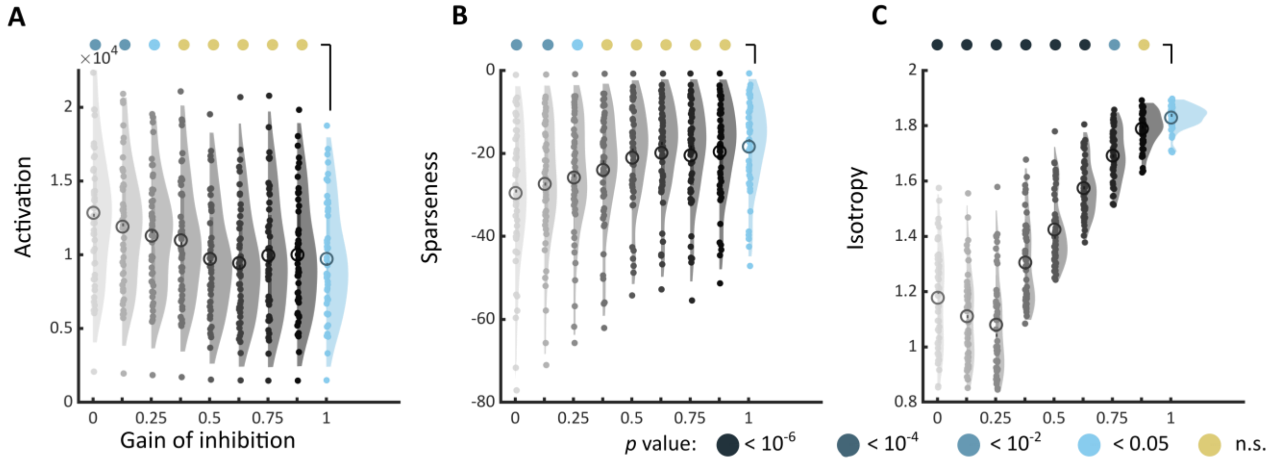

Figure S13. (Counterpart of Figure 5 in the main text for Art 2) Changes in markers of visual discomfort when the balance of excitation over inhibition is modified. Distributions of (A) activation, (B) sparseness, and (C) isotropy metrics for all the stimuli in Art 2 and increasing values of gain for the inhibitory layer. The gain ranged from 0, i.e., no inhibitory activity in the model (top left, light grey distribution), to 1, *i.e.*, reference model (top right, blue distribution), in steps of 0.125. Differences between distributions and the distribution for the reference model were tested using two-sample Kolmogorov-Smirnov tests; p-values are colour coded as in Figure 3 in the main manuscript.

S7. Percentage of stimuli processed beyond 85% discomfort threshold when the gain of inhibition was decreased.

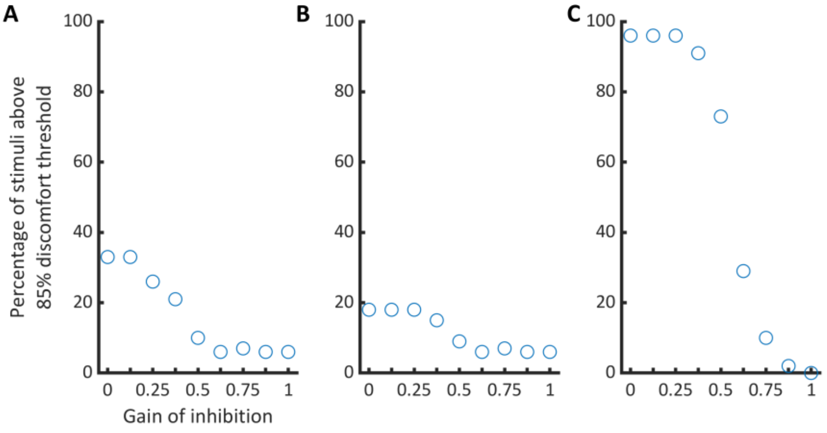

Figure S14. Number of images with a metric value above the threshold associated with 15% most discomfort in the original model as a function of the inhibition in the model for the three markers of discomfort, namely (A) activation, (B) sparseness, and (C) isotropy. The gain of inhibition ranged from 0, i.e., no inhibitory activity in the model (top left, light grey distribution), to 1, *i.e.*, reference model (top right, blue distribution), in steps of 0.125.

S8. Illustration of the 'winner-takes-all' process in orientation columns when inhibition is progressively decreased.

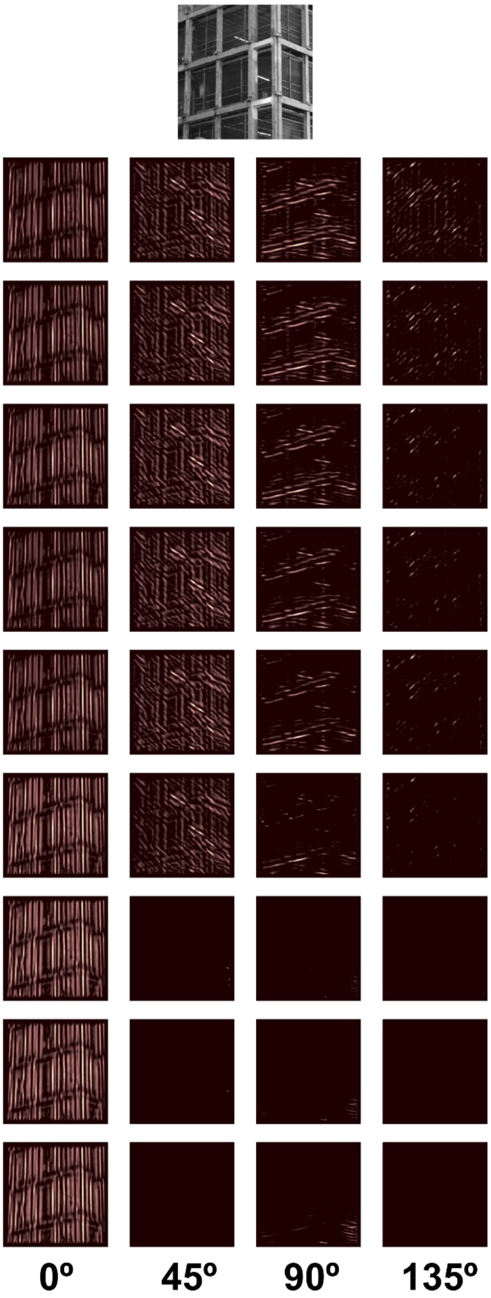

Figure S15. Evolution of the average excitatory activity in the orientation-tuned channels in response of one image in Set 4 when the gain of the inhibitory units in the model decreases. (top, central panel) One image in Set 4. (bottom panels) Average of the excitatory activity in the four orientation planes (respectively sensitive to 0º, left column, 45º, second column, 90º third column and 135º, bottom column) over several membrane time constants (4th to 20th membrane time constant) when the gain of the inhibitory layer is progressively decreased from 1 (default model implementation, top row) to 0 (bottom row) in steps of 0.125, as in Figure 5 in the main text. The lighter the colour, the more activated the cells are. A 'winner-takes-all' process takes place where the orientation 0º take all the activity in the hypercolumns.

S9. Relationship between deviation with respect to 1/f and model activation

We regressed model activation against two different measures of deviation with respect to 1/f. The first measure {Penacchio, 2015 #1658} is built by fitting a natural two-dimensional 1/f cone (i.e., the average of a large number of amplitude spectra of natural images) to the amplitude spectrum of an image and then computing the overall distance between the actual cone and the best fit by summing the 'residuals' of the fitting procedure. Each excess of contrast at any spatial frequency or orientation in the two-dimensional Fourier space can contribute to deviation with respect to the natural 1/f cone. This measure has been shown to be a robust predictor of discomfort {Le, 2017 #1809;Penacchio, 2021 #2217;Penacchio, 2015 #1658;Wilkins, 2018 #2012}. The second measure is the slope of the amplitude spectrum as classically computed by averaging the amplitude across orientations and fitting a regression line in the log-log domain (e.g., {Tolhurst, 1992 #2101}), and used in in (Olman *et al.* 2004; Isherwood, Schira & Spehar 2017). We found strong correlation for all sets but Art 1 for the measure based on computing deviation in the two-dimensional Fourier domain but did not find any correlation for the spectral slope (see table and Figures S16-S19 below; non-significant correlations are highlighted in grey in the table).

**Image set**	**Correlation between model activation and global departure with 2-dimensional amplitude spectrum (Penacchio & Wilkins 2015)**	**Correlation between model activation and spectral slope (Tolhurst, Tadmor & Chao 1992)**
**Architecture 1**	r = 0.52, p = 2.2x10^{-6}<br>ci = [0.33, 0.67]	r = 0.12, p = 0.30, NS<br>ci = [-0.11, 0.34]
**Architecture 1**	r = 0.66, p = 1.9x10^{-10}<br>ci = [0.51, 0.77]	r = 0.23, p = 0.051, NS<br>ci = [-0.001, 0.43]
**Art 1**	r = 0.26, p = 0.071, NS<br>ci = [-0.02, 0.50]	r = -0.19, p = 0.19, NS<br>ci = [-0.44, 0.10]
**Art 2**	r = 0.61, p = 3.2x10^{-6}<br>ci = [0.39, 0.76]	r = 0.06, p = 0.68<br>ci = [-0.22, 0.33]

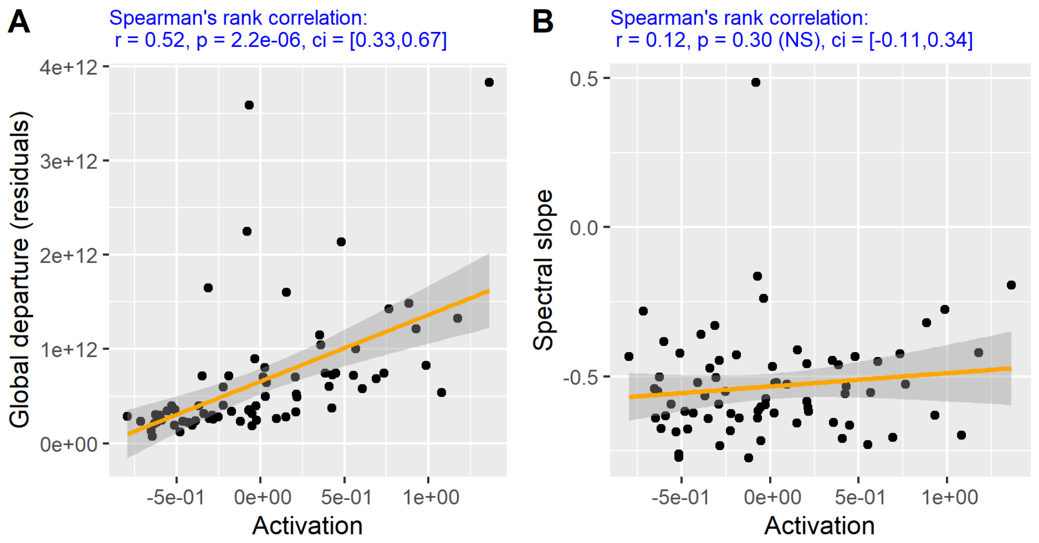

Figure S16. Relationship between model activation level in response to an input image and two measures of deviation with respect to 1/f for set Architecture 1. (A) Deviation from 1/f measured as the deviation between the full 2-dimensional Fourier amplitude spectrum and the average 1/f spectrum for natural scenes (see {Penacchio, 2015 #1658} for details) against model activation. Each point corresponds to a single image in Architecture 1 (N = 74). (B) Spectral slope against model activation. In both panels, the text at the top shows the Spearman's rank correlation between the two metrics.

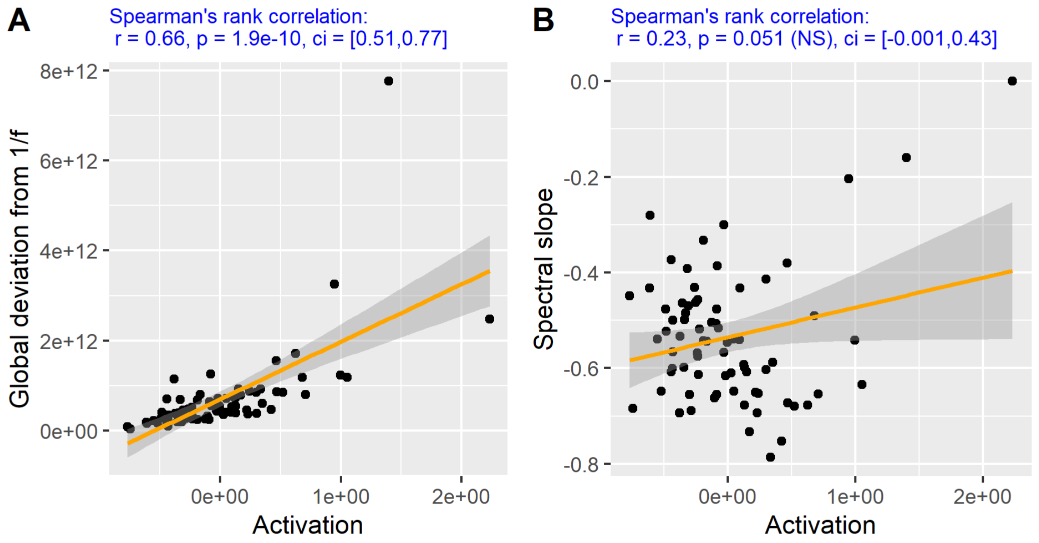

Figure S17. Relationship between model activation level in response to an input image and two measures of deviation with respect to 1/f for set Architecture 2. (A) Deviation from 1/f measured as the deviation between the full 2-dimensional Fourier amplitude spectrum and the average 1/f spectrum for natural scenes (see {Penacchio, 2015 #1658} for details) against model activation. Each point corresponds to a single image in Architecture 2 (N = 74). (B) Spectral slope against model activation. In both panels, the text at the top shows the Spearman's rank correlation between the two metrics.

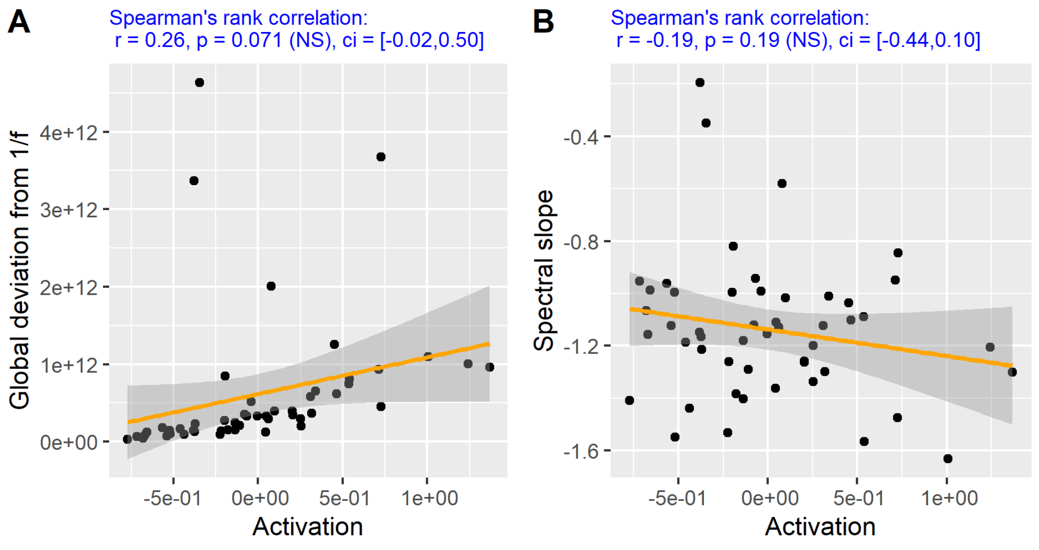

Figure S18. Relationship between model activation level in response to an input image and two measures of deviation with respect to 1/f for set Art 1. (A) Deviation from 1/f measured as the deviation between the full 2-dimensional Fourier amplitude spectrum and the average 1/f spectrum for natural scenes (see {Penacchio, 2015 #1658} for details) against model activation. Each point corresponds to a single image in Art 1 (N = 50). (B) Spectral slope against model activation. In both panels, the text at the top shows the Spearman's rank correlation between the two metrics.

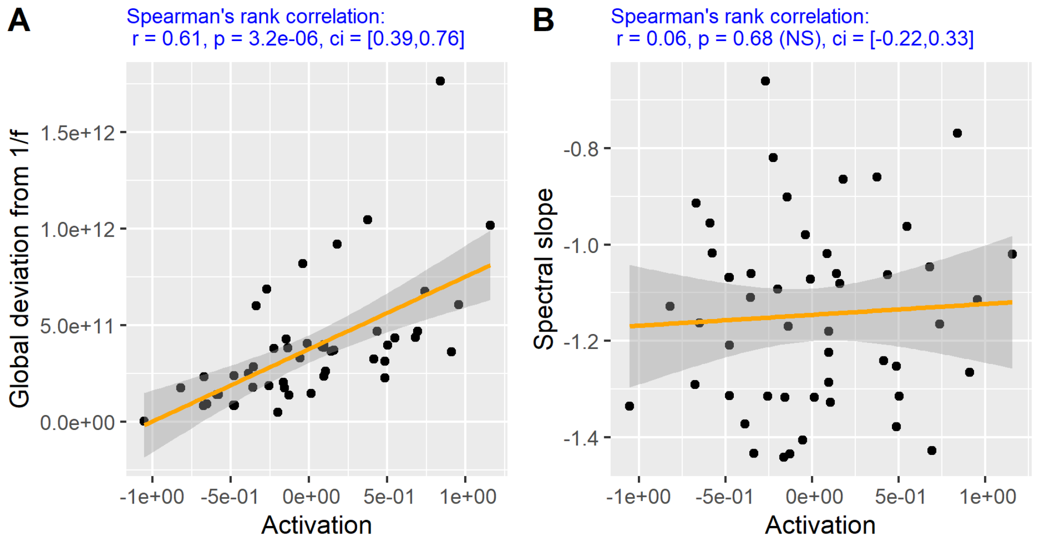

Figure S18. Relationship between model activation level in response to an input image and two measures of deviation with respect to 1/f for set Art 2. (A) Deviation from 1/f measured as the deviation between the full 2-dimensional Fourier amplitude spectrum and the average 1/f spectrum for natural scenes (see {Penacchio, 2015 #1658} for details) against model activation. Each point corresponds to a single image in Art 2 (N = 50). (B) Spectral slope against model activation. In both panels, the text at the top shows the Spearman's rank correlation between the two metrics.

References

Baddeley, R., L. F. Abbott, M. C. A. Booth, F. Sengpiel, T. Freeman, E. A. Wakeman and E. T. Rolls (1997). "Responses of neurons in primary and inferior temporal visual cortices to natural scenes." Proceedings of the Royal Society B-Biological Sciences **264**(1389): 1775-1783.

Berga, D. and X. Otazu (2020). "Modeling bottom-up and top-down attention with a neurodynamic model of V1." Neurocomputing **417**: 270-289.

Berga, D. and X. Otazu (2022). "A Neurodynamic Model of Saliency Prediction in V1." Neural Computation **34**(2): 378-414.

Devalois, R. L., D. G. Albrecht and L. G. Thorell (1982). "Spatial-frequency selectivity of cells in macaque visual-cortex." Vision Research **22**(5): 545-559.

Haider, B., M. R. Krause, A. Duque, Y. Yu, J. Touryan, J. A. Mazer and D. A. McCormick (2010). "Synaptic and Network Mechanisms of Sparse and Reliable Visual Cortical Activity during Nonclassical Receptive Field Stimulation." Neuron **65**(1): 107-121.

Hibbard, P. B. and L. O'Hare (2015). "Uncomfortable images produce non-sparse responses in a model of primary visual cortex." Royal Society open science **2**(2): 140535.

Hurley, N. and S. Rickard (2009). "Comparing Measures of Sparsity." Ieee Transactions on Information Theory **55**(10): 4723-4741.

Kapadia, M. K., M. Ito, C. D. Gilbert and G. Westheimer (1995). "Improvement in visual sensitivity by changes in local context - parallel studies in human observers and in V1 of alert monkeys." Neuron **15**(4): 843-856.

Knierim, J. J. and D. C. Vanessen (1992). "Neuronal responses to static texture patterns in area-V1 of the alert macaque monkey." Journal of Neurophysiology **67**(4): 961-980.

Li, Z. P. (1999). "Visual segmentation by contextual influences via intra-cortical interactions in the primary visual cortex." Network-Computation in Neural Systems **10**(2): 187-212.

Linden, H. and R. W. Berg (2021). "Why Firing Rate Distributions Are Important for Understanding Spinal Central Pattern Generators." Frontiers in Human Neuroscience **15**: 10.

Penacchio, O., X. Otazu and L. Dempere-Marco (2013). "A Neurodynamical Model of Brightness Induction in V1." Plos One **8**(5).

Serre, T., A. Oliva and T. Poggio (2007). "A feedforward architecture accounts for rapid categorization." Proceedings of the National Academy of Sciences of the United States of America **104**(15): 6424-6429.

Serre, T. and M. Riesenhuber (2004). Realistic modeling of simple and complex cell tuning in the HMAX model, and implications for invariant object recognition in cortex, AI Memo 2004-017. CBCL Memo 239. Cambridge, MA, USA, MIT. **239**.

Vinje, W. E. and J. L. Gallant (2000). "Sparse coding and decorrelation in primary visual cortex during natural vision." Science **287**(5456): 1273-1276.

Weliky, M., K. Kandler, D. Fitzpatrick and L. C. Katz (1995). "Patterns of excitation and inhibition evoked by horizontal connections in visual cortex share a common relationship to orientation columns." Neuron **15**(3): 541-552.

Zhang, X. L., L. Zhaoping, T. G. Zhou and F. Fang (2012). "Neural Activities in V1 Create a Bottom-Up Saliency Map." Neuron **73**(1): 183-192.

Zhaoping, L. and K. A. May (2007). "Psychophysical tests of the hypothesis of a bottom-up saliency map in primary visual cortex." Plos Computational Biology **3**(4): 616-633.

Zhaoping, L. and L. Zhe (2015). "Primary Visual Cortex as a Saliency Map: A Parameter-Free Prediction and Its Test by Behavioral Data." Plos Computational Biology **11**(10).
